# Supplementary material for: Equity considerations in outcome measures of the HIV pre-exposure prophylaxis care continuum in high-income countries: a systematic review protocol
Source: BMJ Open. 2021 Feb 4;11(2):e040701. doi: 10.1136/bmjopen-2020-040701 (PMC7868247; doi:10.1136/bmjopen-2020-040701)
Supplement: Supplementary data [file bmjopen-2020-040701supp003.pdf]

Reporting checklist for protocol of a systematic review.

Based on the PRISMA-P guidelines.

Instructions to authors

Complete this checklist by entering the page numbers from your manuscript where readers will find each of the items listed below.

Your article may not currently address all the items on the checklist. Please modify your text to include the missing information. If you are certain that an item does not apply, please write "n/a" and provide a short explanation.

Upload your completed checklist as an extra file when you submit to a journal.

In your methods section, say that you used the PRISMA-Preporting guidelines, and cite them as:

Moher D, Shamseer L, Clarke M, Ghersi D, Liberati A, Petticrew M, Shekelle P, Stewart LA. Preferred Reporting Items for Systematic Review and Meta-Analysis Protocols (PRISMA-P) 2015 statement. Syst Rev. 2015;4(1):1.

		Reporting Item	Page Number
**Title**			
Identification	#1a	Identify the report as a protocol of a systematic review	pg 1, line 2
Update	#1b	If the protocol is for an update of a previous systematic review, identify as such	N/A
**Registration**			
	#2	If registered, provide the name of the registry (such as PROSPERO) and registration number	pg 6, line 200 - 201
**Authors**			
Contact	#3a	Provide name, institutional affiliation, e-mail address of all protocol authors; provide physical mailing address of corresponding author	pg1, line 4-26
Contribution	#3b	Describe contributions of protocol authors and identify the	pg10, line 347-

		guarantor of the review	353
**Amendments**			
	#4	If the protocol represents an amendment of a previously completed or published protocol, identify as such and list changes; otherwise, state plan for documenting important protocol amendments	N/A
**Support**			
Sources	#5a	Indicate sources of financial or other support for the review	pg 10, line 355 - 361
Sponsor	#5b	Provide name for the review funder and / or sponsor	N/A
Role of sponsor or funder	#5c	Describe roles of funder(s), sponsor(s), and / or institution(s), if any, in developing the protocol	N/A
**Introduction**			
Rationale	#6	Describe the rationale for the review in the context of what is already known	pg 4-5, line 103 -170
Objectives	#7	Provide an explicit statement of the question(s) the review will address with reference to participants, interventions, comparators, and outcomes (PICO)	pg. 5-6, line 178 - 189
**Methods**			
Eligibility criteria	#8	Specify the study characteristics (such as PICO, study design, setting, time frame) and report characteristics (such as years considered, language, publication status) to be used as criteria for eligibility for the review	pg. 6-7, line 207-250
Information sources	#9	Describe all intended information sources (such as electronic databases, contact with study authors, trial registers or other grey literature sources) with planned dates of coverage	pg. 8, line 261 - 280
Search strategy	#10	Present draft of search strategy to be used for at least one electronic database, including planned limits, such that it could be repeated	pg. 8, line 276 – 278(and appendix 2)
Study records - data management	#11a	Describe the mechanism(s) that will be used to manage records and data throughout the review	pg. 8, line 282 – 283

Study records - selection process	#11b	State the process that will be used for selecting studies (such as two independent reviewers) through each phase of the review (that is, screening, eligibility and inclusion in meta-analysis)	pg. 8-9, line 282 – 291
Study records - data collection process	#11c	Describe planned method of extracting data from reports (such as piloting forms, done independently, in duplicate), any processes for obtaining and confirming data from investigators	pg. 9, line 292 – 295
Data items	#12	List and define all variables for which data will be sought (such as PICO items, funding sources), any pre-planned data assumptions and simplifications	pg. 9, line 296 – 309
Outcomes and prioritization	#13	List and define all outcomes for which data will be sought, including prioritization of main and additional outcomes, with rationale	pg. 7, line 237 – 244
Risk of bias in individual studies	#14	Describe anticipated methods for assessing risk of bias of individual studies, including whether this will be done at the outcome or study level, or both; state how this information will be used in data synthesis	pg. 9, line 310 – 317
Data synthesis	#15a	Describe criteria under which study data will be quantitatively synthesised	N/A
Data synthesis	#15b	If data are appropriate for quantitative synthesis, describe planned summary measures, methods of handling data and methods of combining data from studies, including any planned exploration of consistency (such as I2, Kendall's τ)	N/A
Data synthesis	#15c	Describe any proposed additional analyses (such as sensitivity or subgroup analyses, meta-regression)	N/A
Data synthesis	#15d	If quantitative synthesis is not appropriate, describe the type of summary planned	pg. 9, line 318 – 331
Meta-bias(es)	#16	Specify any planned assessment of meta-bias(es) (such as publication bias across studies, selective reporting within studies)	N/A
Confidence in cumulative	#17	Describe how the strength of the body of evidence will be assessed (such as GRADE)	pg. 9, 310 – 317

evidence

The PRISMA-P checklist is distributed under the terms of the Creative Commons Attribution License CC-BY 4.0. This checklist was completed on 19. May 2020 using https://www.goodreports.org/, a tool made by the EQUATOR Network in collaboration with Penelope.ai
