# Supplementary material for: Equity considerations in outcome measures of the HIV pre-exposure prophylaxis care continuum in high-income countries: a systematic review protocol
Source: BMJ Open. 2021 Feb 4;11(2):e040701. doi: 10.1136/bmjopen-2020-040701 (PMC7868247; doi:10.1136/bmjopen-2020-040701)
Supplement: Supplementary data [file bmjopen-2020-040701supp002.pdf]

Appendix 2

Systematic Review Search Strategy

1. exp HIV Infections/
2. Pre-Exposure Prophylaxis/
3. Chemoprevention/
4. Anti-HIV Agents/
5. Anti-Retroviral Agents/
6. 1 or 4 or 5
7. 2 or 3
8. 6 and 7
9. ((pre-exposure or preexposure or pre exposure) adj2 prophyla*).tw
10. PrEP.tw
11. ((pre-exposure or preexposure) adj2 chemoprophyla*).tw
12. 10 or 11
13. chemoprophylaxis.tw
14. chemo-prophylaxis.tw
15. chemoprevention.tw
16. chemo-prevention.tw
17. 12 or 13 or 14 or 15 or 16
18. truvada.tw
19. Emtricitabine/
20. Tenofovir/
21. 19 and 20
22. 18 or 21
23. (HIV or Human Immunodeficiency virus).tw
24. 17 and 23
25. 8 or 9 or 22 or 24
26. limit 25 to yr="2012 -Current"
27. limit 26 to (english language and humans)
28. exp Attitude to Health/
29. exp "Treatment Adherence and Compliance"/

30. Program Evaluation/mt, st, sn [Methods, Standards, Statistics & Numerical Data]

31. ((care or care-) adj2 (cascade or continuum)).tw

32. ((prevention or prevention-) adj2 (cascade or continuum)).tw

33. (non-adherence or nonadherence or non adherence).tw

34. (noncompliance or non-compliance or non compliance).tw

35. access.tw

36. adhere*.tw

37. attitude*.tw

38. aware*.tw

39. compliance.tw

40. compliant.tw

41. demand.tw

42. disengage*.tw

43. enable*.tw

44. engage*.tw

45. evaluat*.tw

46. experience*.tw

47. implement*.tw

48. interest.tw

49. knowledge.tw

50. measure*.tw

51. perception*.tw

52. retain*.tw

53. retention.tw

54. uptake.tw

55. 28 or 29 or 30 or 31 or 32 or 33 or 34 or 35 or 36 or 37 or 38 or 39 or 40 or 41 or 42 or 43 or 44 or 45 or 46 or 47 or 48 or 49 or 50 or 51 or 52 or 53 or 54

56. 27 and 55
