# Supplementary material for: Equity considerations in outcome measures of the HIV pre-exposure prophylaxis care continuum in high-income countries: a systematic review protocol
Source: BMJ Open. 2021 Feb 4;11(2):e040701. doi: 10.1136/bmjopen-2020-040701 (PMC7868247; doi:10.1136/bmjopen-2020-040701)
Supplement: Author's manuscript [file bmjopen-2020-040701.draft_revisions.pdf]

BMJ Open

BMJ Open is committed to open peer review. As part of this commitment we make the peer review history of every article we publish publicly available.

When an article is published we post the peer reviewers' comments and the authors' responses online. We also post the versions of the paper that were used during peer review. These are the versions that the peer review comments apply to.

The versions of the paper that follow are the versions that were submitted during the peer review process. They are not the versions of record or the final published versions. They should not be cited or distributed as the published version of this manuscript.

BMJ Open is an open access journal and the full, final, typeset and author-corrected version of record of the manuscript is available on our site with no access controls, subscription charges or pay-per-view fees (http://bmjopen.bmj.com).

If you have any questions on BMJ Open's open peer review process please email info.bmjopen@bmj.com

BMJ Open

Equity considerations in outcome measures of the HIV Pre-Exposure prophylaxis care continuum in high income countries: a systematic review protocol

Journal:	*BMJ Open*
Manuscript ID	bmjopen-2020-040701
Article Type:	Protocol
Date Submitted by the Author:	19-May-2020
Complete List of Authors:	Cabecinha, Melissa; University College London, Research Department of Primary Care and Population Health<br>Solomon, Danielle; University College London, Institute for Global Health<br>Rait, Greta; University College London, Primary Care<br>Saunders, John; National Institute for Health Research , Health Protection Research Unit in Blood Borne and Sexually Transmitted Infections at University College London; University College London, Institute of Global Health, Research Department of Infection and Population Health<br>Mohammed, Hamish; Public Health England, HIV & STI Department<br>McDonagh, Lorraine; University College London, Research Department of Primary Care and Population Health
Keywords:	HIV & AIDS < INFECTIOUS DISEASES, PREVENTIVE MEDICINE, SEXUAL MEDICINE

SCHOLARONE™
Manuscripts

I, the Submitting Author has the right to grant and does grant on behalf of all authors of the Work (as defined in the below author licence), an exclusive licence and/or a non-exclusive licence for contributions from authors who are: i) UK Crown employees; ii) where BMJ has agreed a CC-BY licence shall apply, and/or iii) in accordance with the terms applicable for US Federal Government officers or employees acting as part of their official duties; on a worldwide, perpetual, irrevocable, royalty-free basis to BMJ Publishing Group Ltd ("BMJ") its licensees and where the relevant Journal is co-owned by BMJ to the co-owners of the Journal, to publish the Work in this journal and any other BMJ products and to exploit all rights, as set out in our licence.

The Submitting Author accepts and understands that any supply made under these terms is made by BMJ to the Submitting Author unless you are acting as an employee on behalf of your employer or a postgraduate student of an affiliated institution which is paying any applicable article publishing charge ("APC") for Open Access articles. Where the Submitting Author wishes to make the Work available on an Open Access basis (and intends to pay the relevant APC), the terms of reuse of such Open Access shall be governed by a Creative Commons licence – details of these licences and which Creative Commons licence will apply to this Work are set out in our licence referred to above.

Other than as permitted in any relevant BMJ Author's Self Archiving Policies, I confirm this Work has not been accepted for publication elsewhere, is not being considered for publication elsewhere and does not duplicate material already published. I confirm all authors consent to publication of this Work and authorise the granting of this licence.

For peer review only

EQUITY CONSIDERATIONS IN OUTCOME MEASURES OF THE HIV PRE-EXPOSURE PROPHYLAXIS

CARE CONTINUUM IN HIGH INCOME COUNTRIES: A SYSTEMATIC REVIEW PROTOCOL

Melissa Cabecinha[1]

(m.cabecinha@ucl.ac.uk)

Danielle Solomon[2]

(danielle.solomon@ucl.ac.uk)

Greta Rait[1,3]

(g.rait@ucl.ac.uk)

John Saunders[3,4]

(john.saunders@ucl.ac.uk)

Hamish Mohammed[5,6]

(hamish.mohammed@phe.gov.uk)

&

Lorraine K. McDonagh[1,3]

(l.mcdonagh@ucl.ac.uk)

[1]Research Department of Primary Care and Population Health, University College London,

[2]Institute for Global Health, University College London

[3]National Institute for Health Research Health Protection Research Unit in Blood Borne and Sexually Transmitted Infections at University College London

[4]UCL Centre for Clinical Research in Infection and Sexual Health, Institute for Global Health, University College London

For peer review only

[5]Blood Safety, Hepatitis, Sexually Transmitted Infections (STI) and HIV Division, National Infection Service, Public Health England

[6]Infection & Population Health, Institute for Global Health, University College, London

CORRESPONDENCE TO: Ms Melissa Cabecinha; m.cabecinha@@ucl.ac.uk; Research Department of Primary Care and Population Health, Upper third floor, UCL Medical School (Royal Free Campus), Rowland Kill Street, London, NW3 2PF

KEYWORDS: HIV Pre-Exposure Prophylaxis; HIV prevention; health service evaluation; health equity

WORD COUNT: 3,011 words

For peer review only

ABSTRACT

Introduction

HIV Pre-exposure prophylaxis (PrEP) is an effective intervention to reduce acquisition of HIV. PrEP provision has increased in recent years, however, it is not known whether PrEP implementation has been equitably implemented across all risk groups, particularly groups experiencing high levels of health inequity. A PrEP care-continuum (PCC) has been proposed to evaluate the success of PrEP implementation programmes, but the extent to which health equity characteristics are currently taken into account in the PCC has not been described. The objectives of this proposed systematic review are to 1) identify and collate outcome measure definitions for the main stages of the PCC (awareness, acceptability, uptake, adherence, and retention), 2) describe how equity characteristics are considered in outcome definitions of the PCC, and 3) describe data sources for capturing equity characteristics.

Methods and analysis

Quantitative studies published after 2012 will be included. Five databases (MEDLINE, PubMed, Embase, CINHAL, ASSIA) will be searched to identify English language publications that include an outcome measure definition of at least one of the five main stages of the PCC. Risk of bias will be assessed using the Effective Public Health Practice Project Quality Assessment Tool for Quantitative Studies. Data on outcome measure definitions and equity characteristics will be extracted. Results will be presented in a narrative synthesis and all findings will be reported in accordance with the Preferred Reporting Items for Systematic Reviews and Meta-Analyses guidelines.

Ethics and dissemination

For peer review only - http://bmjopen.bmj.com/site/about/guidelines.xhtml

Ethical approval is not required. The results will be disseminated via submission for publication to a peer-reviewed journal when complete. The review findings will have relevance to healthcare professionals, policymakers and commissioners in informing how to best evaluate PrEP implementation programmes and inform new implementation strategies for vulnerable and less advantaged populations.

For peer review only

ARTICLE SUMMARY

Strengths and limitations of this study

- This systematic review will be the first to collate outcome measures for the HIV Pre-Exposure Prophylaxis (PrEP) Care Continuum from real-world settings and investigate how equity characteristics are considered within existing measures.
- The findings from this review will help inform evaluation and monitoring of PrEP programmes and how to view these evaluations through an equity lens.
- This protocol has been written in accordance with published PRISMA-P guidelines, and the review will be reported in accordance with the PRISMA statement guidelines and the PRISMA-E 2012 equity extension.
- This review will not provide specific information on the effectiveness of existing PrEP programmes at reducing health inequity or population vulnerability.

For peer review only - http://bmjopen.bmj.com/site/about/guidelines.xhtml

For peer review only

INTRODUCTION

Oral HIV Pre-Exposure prophylaxis (HIV PrEP, hereafter referred to as PrEP) is a highly effective biomedical intervention for reducing HIV acquisition, and is recommended by the World Health Organisation (WHO) as part of a comprehensive approach to HIV prevention[1]. The efficacy of PrEP has been demonstrated in clinical trials[2,3], and PrEP provision has been increasing globally in recent years[4]. However, questions remain as to whether real-world implementation strategies are reaching some high-risk and vulnerable populations[5], particularly those populations experiencing high levels of health inequity.

Health Inequity and HIV

Health inequities are disparities in health status or access to healthcare that are considered unnecessary, avoidable, unfair and unjust[6]. Health equity is an important consideration in HIV prevention, as HIV disproportionately affects marginalised populations. Gender and sexual minorities[7–9], ethnic minorities[7,10], vulnerable populations such as people who inject drugs[11], people experiencing housing instability[12] and other social disadvantages[13] are at a greater risk of HIV acquisition compared to the general population. These categories are not mutually exclusive; an individual may identify with multiple categories concurrently[14] and the experience of belonging to multiple marginalized groups may contribute to an increased risk of acquiring HIV[15]. HIV prevention programmes, including PrEP provision programmes,

For peer review only - http://bmjopen.bmj.com/site/about/guidelines.xhtml

must address the needs of marginalised populations and communities in order to have a sustained impact on the decline of HIV transmission[11].

Measureable Outcomes for PrEP Implementation

A PrEP Care Continuum (PCC) has been proposed[16,17] to evaluate the implementation of PrEP. The main stages of the PCC are as follows: awareness (knowledge of PrEP and indication of relevance), acceptability (whether an individual views PrEP as an acceptable tool for prevention), uptake (engagement in PrEP related care and initiation of PrEP), and adherence (retention in PrEP related care and adherence to treatment). Although the PCC provides a useful framework, in practice the outcomes have been reported with varying definitions. For example, in evaluating the “uptake” stage of the continuum, a study of Veterans in the United States reports uptake as the absolute number of people initiating on PrEP within a given time-frame[18], whereas The South Africa Program defines uptake within a given programme as the proportion initiating PrEP after both a negative test for HIV and an offer of oral PrEP[19]. In England, the PrEP Impact Trial defines uptake as the proportion initiating oral PrEP among those meeting the eligibility criteria in participating sexual health clinics[20]. A common definition of uptake has been proposed for monitoring PrEP delivery to adolescent girls and young women in sub-Saharan Africa (“the number who initiate oral PrEP among those offered PrEP”)[21] and consistent definitions for the outcome measures of the PCC would be beneficial for comparing the relative success of PrEP provision programmes.

Health Inequity and PrEP

These measures are important for evaluating the overall success of PrEP programmes, identifying populations with low engagement, and ensuring that programmes do not increase health inequity among at-risk populations. The latter can be achieved, in part, by considering health equity characteristics when evaluating PrEP provision programmes. Structural and environmental factors that contribute to discrepancies in HIV rates have been shown to affect HIV treatment uptake and adherence[22,23], uptake of HIV prevention strategies in general[23] and for PrEP specifically. For example, lower levels of education, anticipated stigma, and higher PrEP cost have been associated with lower willingness to take PrEP[24]. Low adherence to PrEP has been associated with competing survival needs (e.g. financial instability, housing) [25] and substance use[26]. It is likely that other structural, social and environmental factors influence engagement at each stage of the PCC, such as those summarised by the PROGRESS-Plus framework[27,28].

The PROGRESS-Plus framework describes a sample of characteristics that drive disparities in health[28,29]. The acronym PROGRESS refers to Place of residence (e.g., rural/urban/inner city), Race/ethnicity/culture/language, Occupation, Gender/sex, Religion, Education, Socio-economic status, and Social capital. The “Plus” suffix includes additional categories that can attract discrimination for consideration: personal characteristics (such as age or having a disability), features of a relationship (such as being excluded from school or having unpaid care responsibilities), and time dependent relationships (such as leaving hospital or respite care).

For peer review only - http://bmjopen.bmj.com/site/about/guidelines.xhtml

Currently, the extent to which characteristics influencing health equity, such as those in the PROGRESS-Plus framework, are being investigated in outcome measures of the PCC is not well characterised. The evaluation of health equity within PrEP implementation programmes could be improved by collating current approaches for evaluating outcome measures of the PCC and existing approaches to considering health equity within them.

In order to minimise heterogeneity, this review will focus on outcome measures of the PCC in high-income countries. Although factors influencing health equity in high-income countries are likely to be similar to those in low- and middle-income countries, how PrEP programmes are monitored, and the way vulnerable populations experience health inequity, may differ between these settings.

Aim

This paper is a protocol for a systematic review that aims to identify and collate definitions for outcome measures of the PCC and describe how, and the extent to which, characteristics that influence health equity are taken into account in these measures in high income countries.

Objectives

The objectives of this proposed systematic review are:

1) To identify and collate definitions of outcome measures used to evaluate the main stages of the PCC (awareness, acceptability, uptake, adherence and retention) in high income countries.

2) To explore whether and how studies reporting outcome measures of the PCC consider the effects of health equity characteristics when reporting PrEP awareness, acceptability, uptake, adherence and retention.

3) To describe the sources of data and methods used for capturing equity characteristics (e.g., self-reported survey data, administrative data, linked datasets)

This review aims to describe how outcome measures for the PCC are defined and to describe how health equity characteristics are currently taken into account for these measures. This review is not intended to summarise the effectiveness of PrEP programmes at reducing health inequity or population vulnerability.

METHOD DESIGN

Protocol and registration

This protocol was reported according to the Preferred Reporting Items for Systematic Review and Meta-Analysis Protocols (PRIMSA-P) 2015 statement[30] (appendix 1) and has been registered with the PROSPERO international prospective register of systematic reviews (Prospero registration number: CRD42020169779, available at: https://www.crd.york.ac.uk/prospero/display_record.php?RecordID=169779).

For peer review only - http://bmjopen.bmj.com/site/about/guidelines.xhtml

The review will be conducted and reported in accordance with the PRISMA statement guidelines[31] and the PRISMA-E 2012 equity extension[32].

Eligibility criteria

To be included in the review, papers will have to meet the following Population, Intervention, Context, Outcomes and Study design elements:

1. Population

Inclusion criteria: People resident in high-income countries with a national PrEP provision programme or demonstration/implementation project, regardless of sexual orientation/identity, age, gender identity, or occupation.

Exclusion criteria: People resident in countries without a national PrEP provision program or demonstration/implementation project; people resident in low- or middle-income countries.

2. Intervention

The issue to be reviewed is outcome measures for five stages of the PCC (awareness, acceptability, uptake, adherence and retention). Definitions of outcome measures for the PCC will be collated and reviewed, rather than the reported outcomes. For example, in studies reporting PrEP awareness, the definition of awareness and the metrics used to determine awareness are relevant to this review, whereas the reported level of awareness is not. As such, studies which include descriptions of how the main stages of the PCC will be evaluated but do not report these outcomes (e.g. study protocols) will be included.

Inclusion criteria: Studies reporting an outcome measure definition for at least one stage of the PCC in a real-world setting.

Exclusion Criteria: Studies that do not report an outcome measure definition for the PCC, randomised controlled trials (RCTs).

3. Context

Inclusion criteria: Studies conducted in high income countries, as defined by the World Bank country classifications[33]. Inequity is a relative concept, and inequities experienced by vulnerable populations in lower and middle-income countries may differ from that experienced by vulnerable populations in high-income countries with well-resourced healthcare settings.

Exclusion criteria: Studies conducted in low- or middle- income countries.

4. Outcomes

Primary outcomes: Studies will be included if they report at least one outcome measure definition for the PCC relating to PrEP provision in a “real-world” setting, i.e. an implementation trial, demonstration project or national programme. Studies only reporting absolute numbers for stages of the PCC will not be included. Study protocols, policy frameworks and clinical guidelines will be included if they include definitions of outcome

For peer review only - http://bmjopen.bmj.com/site/about/guidelines.xhtml

measures in relation to a real-world program; for example definitions for how the numerator (e.g. the number of people with high adherence to PrEP) and denominator (e.g. the number of people taking PrEP) will be defined in a measure of PrEP adherence.

Secondary outcomes: Data on whether factors influencing health equity (defined by the PROGRESS-Plus framework) are considered for the description of baseline characteristics of participants and/or when reporting measurable outcomes for the PCC.

5. Study Design

Inclusion criteria: Quantitative research studies including non-experimental observational studies (e.g. cohort, cross-sectional, and longitudinal studies) and abstracts from key international HIV/STI conferences (e.g. HIV/STI World Congress, International AIDS society, Conference on Retroviruses and Opportunistic Infections). Mixed method studies will be included providing a quantified outcome measure of the PCC is reported. Studies resulting from demonstration and/or implementation trials will also be included.

Exclusion Criteria: Qualitative studies, RCTs exclusively reporting on PrEP efficacy/safety (i.e. pharmacological studies), review articles, case studies, studies not involving oral emtricitabine/tenofovir disoproxil fumarate as PrEP, and commentary or opinion pieces that do not contain a definition for an outcome measure of the PCC.

Information sources

Electronic searches

To identify studies, the following electronic databases will be searched: MEDLINE (via Ovid), EMBASE (via Ovid), PubMed, Cumulative Index to Nursing and Allied Health Literature (CINAHL) (via EBSCO Host), and Applied Social Sciences Index and Abstracts (ASSIA) (via ProQuest).

Search strategy

Restrictions on English language articles as well as for articles published between 2012 (the year the United States Federal Drug Administration approved Truvada [emtricitabine/tenofovir disoproxil] fumarate for PrEP use[34]) and present will be applied.

Medical Subject headings (MeSH), subject headings and keyword lists will be created by using language that describes 1) oral PrEP and 2) the following outcome measures of the PCC: awareness, acceptability, uptake, adherence and retention. Boolean combinations will be employed to refine the search strategy. Several key articles will be identified in the preliminary searches and used to test the sensitivity of the search strategy.

The search strategy that will be used to search MEDLINE (via the Ovid platform) is presented in appendix 2. Search terms will be modified for databases where the indexing of subject headings differs from the terms used in MEDLINE.

For peer review only - http://bmjopen.bmj.com/site/about/guidelines.xhtml

The references of articles meeting the inclusion criteria will be searched to identify any relevant papers missed in the electronic searches.

Study selection

All citations retrieved from the electronic searches will be imported into an EndNote database. After duplicates are removed, the remaining studies will be screened by title and abstract by one reviewer (MC), with a random sample independently reviewed by a second reviewer (DS). Reviewers will compare their decisions throughout the process and discuss all references where there was a disagreement. A third reviewer (LMD) will be asked to adjudicate where discrepancies cannot be resolved. Studies remaining after the initial title and abstract screen will undergo full text screening. A flow diagram will be produced to show selection process, according to PRISMA guidelines[35], with explanation for those excluded at each stage.

Data extraction and management

Following the full-text screening, data will be extracted from all studies that meet the inclusion criteria. Data will be extracted into a pilot-tested, standardised form by one reviewer (MC) with a random sample checked by a second reviewer (DS). Any discrepancies will be resolved by discussion or adjudication by a third reviewer (LMD) where necessary.

All studies that meet the inclusion criteria will be described in terms of:

- Bibliographic information (first author, year of study, country of study)
- Study aims and design (including study population, sample size, and data collection methods)
- Participants, demographic characteristics and number of baseline PROGRESS-Plus equity characteristics reported
- Outcome measure definitions (for PrEP awareness, acceptability, uptake, adherence, and retention) and reported outcomes.
- Whether the study considered PROGRESS-Plus equity characteristics when measuring outcomes of the PCC.

For studies that consider PROGRESS-Plus equity characteristics in outcome measures, further data extraction detailing how the PROGRESS-Plus characteristics are expressed, and additional information on the study methods and study results will be extracted into an expanded data collection form.

Risk of bias assessment of included studies

The quality of each paper included in the data extraction stage will be assessed independently by two reviewers, using the Effective Public Health Practice Project (EPHPP) "Quality Assessment Tool for Quantitative Studies"[36]. Individual studies will be categorised as "strong", "moderate", or "weak" in the following domains: selection bias, study design, confounders, blinding, data collection method, and withdrawals and drop outs. Studies will also be given a global score to indicate overall quality. Any discrepancies between the two reviewers will be resolved by consensus, or by a third reviewer if necessary.

For peer review only - http://bmjopen.bmj.com/site/about/guidelines.xhtml

Data synthesis and analysis

A narrative synthesis of results will be conducted. Individual study characteristics and outcomes will be summarised and presented in an evidence table.

To assess the extent to which studies consider the effects of PROGRESS-Plus equity characteristics in the outcome measures of the PCC, a two-stage analysis will be carried out. At the first stage, data will be presented on PCC outcome measure definitions and whether the study considers any of the PROGRESS-Plus equity characteristics for the description of baseline characteristics of study participants. The second stage we will describe whether the study considered PROGRESS-Plus characteristics in outcome measures of the PrEP continuum, and which methods were used (e.g. consideration as confounders by adjusting in multivariable analysis, as effect modifiers in stratified analysis, using interaction terms in multivariable analysis etc.). The ways in which inequities are expressed (i.e. whether absolute or relative) will also be reported, as the decision about whether to measure inequity in relative or absolute terms may influence their interpretation.

DISCUSSION AND DISSEMINATION

To the best of the author's knowledge, this is the first systematic review to collate definitions for outcome measures of the PCC and describe the extent to (and ways in which) equity characteristics are considered in these outcomes. The findings will have relevance to healthcare professionals, policymakers and commissioners in informing how to evaluate PrEP implementation programmes, and how to view these evaluations through an equity lens to optimise HIV prevention strategies and inform new implementation strategies for vulnerable and less advantaged populations.

When complete, the review results will be submitted for publication in a peer-reviewed journal and submitted to be presented at national and international conferences (where eligible). This protocol was informed by input from a Patient and Public Involvement (PPI) representative, and the findings, dissemination strategy, and lay summary will be discussed with a PPI panel.

Authors contributions

MC and LMD designed the study. MC developed and refined the study protocol with contributions from all co-authors (DS, JS, GR, HM and LMD). MC prepared the manuscript. MC will undertake data collection (literature search, data extraction), analysis, interpretation and report writing. All co-investigators will contribute to the design, analysis, interpretation and report writing. All authors read and approved the final manuscript.

Funding Statement

MC is funded by the Medical Research Council Doctoral Training Partnership (grant number: MR/N013867/1). The research is funded by the National Institute for Health Research Health Protection Research Unit (NIHR HPRU) in Blood Borne and Sexually Transmitted Infections at University College London in partnership with Public Health England (reference

For peer review only - http://bmjopen.bmj.com/site/about/guidelines.xhtml

number: NIHR200911). The views expressed in this publication are those of the authors and not necessarily those of the NHS, the Medical Research Council, the National Institute for Health Research, the Department of Health or Public Health England.

Competing interests statement:

None declared

For peer review only

REFERENCES

1. World Health Organization. *Guideline on When To Start Antiretroviral Therapy and on Pre-Exposure Prophylaxis for HIV*. (2015).

2. Grant, R. M. *et al.* Preexposure Chemoprophylaxis for HIV Prevention in Men Who Have Sex with Men. *N. Engl. J. Med.* **363**, 2587–2599 (2010).

3. Baeten, J. M. *et al.* Antiretroviral Prophylaxis for HIV Prevention in Heterosexual Men and Women. *N. Engl. J. Med.* **367**, 399–410 (2012).

4. Country Updates – PrEPWatch. https://www.prepwatch.org/in-practice/country-updates/.

5. Pyra, M. N. *et al.* Global implementation of PrEP for HIV prevention: setting expectations for impact. *J. Int. AIDS Soc.* **22**, (2019).

6. Marmot, M., Friel, S., Bell, R., Houweling, T. A. & Taylor, S. Closing the gap in a generation: health equity through action on the social determinants of health. *Lancet* **372**, 1661–1669 (2008).

7. Public Health England. Trends in new HIV diagnoses and people receiving HIV-related care in the United Kingdom: data to the end of December 2017. *Heal. Prot. Rep. Adv. Access Rep. [v2]* **volume 12**, (2018).

8. Centers for Disease Control and Prevention. HIV and Young Men Who Have Sex With Men. *Natl. Cent. HIV/AIDS, VIral Hepatitis, STD TB Prev.* 1–4 (2014).

9. Mayer, K. H., Grinsztejn, B. & El-Sadr, W. M. Transgender People and HIV Prevention. *JAIDS J. Acquir. Immune Defic. Syndr.* **72**, S207–S209 (2016).

For peer review only - http://bmjopen.bmj.com/site/about/guidelines.xhtml

10. Grossman, C. I., Purcell, D. W., Rotheram-Borus, M. J. & Veniegas, R. Opportunities for HIV combination prevention to reduce racial and ethnic health disparities. *Am. Psychol.* **68**, 237–246 (2013).

11. UNAIDS. *Miles to go: closing gaps, breaking barriers, fighting injustices*. (2018).

12. Elifson, K. W., Sterk, C. E. & Theall, K. P. Safe living: The impact of unstable housing conditions on HIV risk reduction among female drug users. *AIDS Behav.* **11**, (2007).

13. Pellowski, J. A., Kalichman, S. C., Matthews, K. A. & Adler, N. A pandemic of the poor: social disadvantage and the U.S. HIV epidemic. *Am. Psychol.* **68**, 197–209.

14. Rapid Response Service. *Intersectionality in HIV and Other Health-Related Research*. www.health.gov.on.ca/en/public/ (2013).

15. Collins, P. Y., von Unger, H. & Armbrister, A. Church ladies, good girls, and locas: Stigma and the intersection of gender, ethnicity, mental illness, and sexuality in relation to HIV risk. *Soc. Sci. Med.* **67**, 389–397 (2008).

16. Kelley, C. F. *et al.* Applying a PrEP Continuum of Care for Men Who Have Sex with Men in Atlanta, Georgia. *Clin. Infect. Dis.* **61**, 1590–1597 (2015).

17. Nunn, A. S. *et al.* Defining the HIV pre-exposure prophylaxis care continuum. *AIDS* **31**, 731–734 (2017).

18. Chartier, M. *et al.* Accessibility and Uptake of Pre-Exposure Prophylaxis for HIV Prevention in the Veterans Health Administration. *Fed Pr.* **35**, S42-s48 (2018).

19. South African National Department of Health (NDoH). Pre-exposure prophylaxis implementation in South Africa. in *Presented at the 19th Annual International Conference on AIDS and STIs in Africa (ICASA)* (2017).

20. PrEP Impact Trial. A pragmatic health technology assessment of PrEP and implementation. Trial Protocol. v3.0. https://www.prepimpacttrial.org.uk/protocol.

21. Dunbar, M. S. *et al.* Understanding and measuring uptake and coverage of oral pre-exposure prophylaxis delivery among adolescent girls and young women in sub-Saharan Africa. *Sexual Health* vol. 15 513–521 (2018).

22. Duff, P. *et al.* Barriers to Viral Suppression among Female Sex Workers: Role of Structural and Intimate Partner Dynamics. *J. Acquir. Immune Defic. Syndr.* **73**, 83–90 (2016).

23. Gari, S. *et al.* Access to HIV/AIDS care: A systematic review of socio-cultural determinants in low and high income countries. *BMC Health Services Research* vol. 13 (2013).

24. Yi, S. *et al.* Awareness and willingness to use HIV pre-exposure prophylaxis among men who have sex with men in low- and middle-income countries: A systematic review and meta-analysis: A. *Journal of the International AIDS Society* vol. 20 (2017).

25. Janet, J. M. *et al.* Adherence to PrEP among young men who have sex with men participating in a sexual health services demonstration project in Alameda County, California. *J Acquir Immune Defic Syndr* (2019) doi:10.1097/qai.0000000000002051.

26. Zucker, J. *et al.* Predictors of Disengagement in Care for Individuals Receiving Pre-Exposure Prophylaxis (PrEP). *J Acquir Immune Defic Syndr* (2019) doi:10.1097/qai.0000000000002054.

27. O'Neill, J. *et al.* Applying an equity lens to interventions: Using PROGRESS ensures consideration of socially stratifying factors to illuminate inequities in health. *J. Clin. Epidemiol.*

For peer review only - http://bmjopen.bmj.com/site/about/guidelines.xhtml

67, 56–64 (2014).

28. Kavanagh, J., Oliver, S. & Lorenc, T. Reflections on developing and using PROGRESS-Plus. *Equity Updat.* **2**, 1–3 (2008).

29. Evans, T. & Brown, H. Road traffic crashes: operationalizing equity in the context of health sector reform. *Inj. Control Saf. Promot.* **10**, 11–2.

30. Moher, D. *et al.* Preferred reporting items for systematic review and meta-analysis protocols (PRISMA-P) 2015 statement. *Rev. Esp. Nutr. Humana y Diet.* **20**, 148–160 (2016).

31. Liberati, A. *et al.* The PRISMA statement for reporting systematic reviews and meta-analyses of studies that evaluate healthcare interventions: explanation and elaboration. *BMJ* **339**, (2009).

32. Welch, V. *et al.* PRISMA-Equity 2012 Extension: Reporting Guidelines for Systematic Reviews with a Focus on Health Equity. *PLoS Med.* **9**, (2012).

33. WorldBank. World Bank Country and Lending Groups – World Bank Data Help Desk. https://datahelpdesk.worldbank.org/knowledgebase/articles/906519 (2019).

34. FDA. Truvada for PrEP Fact Sheet : Ensuring Safe and Proper Use. 1–2 (2012).

35. Shamseer, L. *et al.* Preferred reporting items for systematic review and meta-analysis protocols (prisma-p) 2015: Elaboration and explanation. *BMJ (Online)* vol. 350 (2015).

36. Quality Assessment Tool for Quantitative Studies. https://www.ephpp.ca/quality-assessment-tool-for-quantitative-studies/.

For peer review only

For peer review only - http://bmjopen.bmj.com/site/about/guidelines.xhtml

PRISMA-P (Preferred Reporting Items for Systematic review and Meta-Analysis Protocols) 2015 checklist: recommended items to address in a systematic review protocol*

Section and topic	Item No	Checklist item
**ADMINISTRATIVE INFORMATION**		
Title:		
Identification	1a	Identify the report as a protocol of a systematic review
Update	1b	If the protocol is for an update of a previous systematic review, identify as such
Registration	2	If registered, provide the name of the registry (such as PROSPERO) and registration number
Authors:		
Contact	3a	Provide name, institutional affiliation, e-mail address of all protocol authors; provide physical mailing address of corresponding author
Contributions	3b	Describe contributions of protocol authors and identify the guarantor of the review
Amendments	4	If the protocol represents an amendment of a previously completed or published protocol, identify as such and list changes; otherwise, state plan for documenting important protocol amendments
Support:		
Sources	5a	Indicate sources of financial or other support for the review
Sponsor	5b	Provide name for the review funder and/or sponsor
Role of sponsor or funder	5c	Describe roles of funder(s), sponsor(s), and/or institution(s), if any, in developing the protocol
**INTRODUCTION**		
Rationale	6	Describe the rationale for the review in the context of what is already known
Objectives	7	Provide an explicit statement of the question(s) the review will address with reference to participants, interventions, comparators, and outcomes (PICO)
**METHODS**		
Eligibility criteria	8	Specify the study characteristics (such as PICO, study design, setting, time frame) and report characteristics (such as years considered, language, publication status) to be used as criteria for eligibility for the review
Information sources	9	Describe all intended information sources (such as electronic databases, contact with study authors, trial registers or other grey literature sources) with planned dates of coverage
Search strategy	10	Present draft of search strategy to be used for at least one electronic database, including planned limits, such that it could be repeated
Study records:		
Data management	11a	Describe the mechanism(s) that will be used to manage records and data throughout the review

For peer review only

Selection process	11b	State the process that will be used for selecting studies (such as two independent reviewers) through each phase of the review (that is, screening, eligibility and inclusion in meta-analysis)
Data collection process	11c	Describe planned method of extracting data from reports (such as piloting forms, done independently, in duplicate), any processes for obtaining and confirming data from investigators
Data items	12	List and define all variables for which data will be sought (such as PICO items, funding sources), any pre-planned data assumptions and simplifications
Outcomes and prioritization	13	List and define all outcomes for which data will be sought, including prioritization of main and additional outcomes, with rationale
Risk of bias in individual studies	14	Describe anticipated methods for assessing risk of bias of individual studies, including whether this will be done at the outcome or study level, or both; state how this information will be used in data synthesis
Data synthesis	15a	Describe criteria under which study data will be quantitatively synthesised
	15b	If data are appropriate for quantitative synthesis, describe planned summary measures, methods of handling data and methods of combining data from studies, including any planned exploration of consistency (such as I^2, Kendall's τ)
	15c	Describe any proposed additional analyses (such as sensitivity or subgroup analyses, meta-regression)
	15d	If quantitative synthesis is not appropriate, describe the type of summary planned
Meta-bias(es)	16	Specify any planned assessment of meta-bias(es) (such as publication bias across studies, selective reporting within studies)
Confidence in cumulative evidence	17	Describe how the strength of the body of evidence will be assessed (such as GRADE)

*** It is strongly recommended that this checklist be read in conjunction with the PRISMA-P Explanation and Elaboration (cite when available) for important clarification on the items. Amendments to a review protocol should be tracked and dated.** **The copyright for PRISMA-P (including checklist) is held by the PRISMA-P Group and is distributed under a Creative Commons Attribution Licence 4.0.**

From: Shamseer L, Moher D, Clarke M, Ghersi D, Liberati A, Petticrew M, Shekelle P, Stewart L, PRISMA-P Group. Preferred reporting items for systematic review and meta-analysis protocols (PRISMA-P) 2015: elaboration and explanation. BMJ. 2015 Jan 2;349(jan02 1):g7647.

For peer review only

Appendix 2

Systematic Review Search Strategy

1. exp HIV Infections/
2. Pre-Exposure Prophylaxis/
3. Chemoprevention/
4. Anti-HIV Agents/
5. Anti-Retroviral Agents/
6. 1 or 4 or 5
7. 2 or 3
8. 6 and 7
9. ((pre-exposure or preexposure or pre exposure) adj2 prophyla*).tw
10. PrEP.tw
11. ((pre-exposure or preexposure) adj2 chemoprophyla*).tw
12. 10 or 11
13. chemoprophylaxis.tw
14. chemo-prophylaxis.tw
15. chemoprevention.tw
16. chemo-prevention.tw
17. 12 or 13 or 14 or 15 or 16
18. truvada.tw
19. Emtricitabine/
20. Tenofovir/
21. 19 and 20
22. 18 or 21
23. (HIV or Human Immunodeficiency virus).tw
24. 17 and 23
25. 8 or 9 or 22 or 24
26. limit 25 to yr="2012 -Current"
27. limit 26 to (english language and humans)
28. exp Attitude to Health/
29. exp "Treatment Adherence and Compliance"/

For peer review only

30. Program Evaluation/mt, st, sn [Methods, Standards, Statistics & Numerical Data]

31. ((care or care-) adj2 (cascade or continuum)).tw

32. ((prevention or prevention-) adj2 (cascade or continuum)).tw

33. (non-adherence or nonadherence or non adherence).tw

34. (noncompliance or non-compliance or non compliance).tw

35. access.tw

36. adhere*.tw

37. attitude*.tw

38. aware*.tw

39. compliance.tw

40. compliant.tw

41. demand.tw

42. disengage*.tw

43. enable*.tw

44. engage*.tw

45. evaluat*.tw

46. experience*.tw

47. implement*.tw

48. interest.tw

49. knowledge.tw

50. measure*.tw

51. perception*.tw

52. retain*.tw

53. retention.tw

54. uptake.tw

55. 28 or 29 or 30 or 31 or 32 or 33 or 34 or 35 or 36 or 37 or 38 or 39 or 40 or 41 or 42 or 43 or 44 or 45 or 46 or 47 or 48 or 49 or 50 or 51 or 52 or 53 or 54

56. 27 and 55

For peer review only

BMJ Open

Equity considerations in outcome measures of the HIV Pre-Exposure prophylaxis care continuum in high income countries: a systematic review protocol

Journal:	*BMJ Open*
Manuscript ID	bmjopen-2020-040701.R1
Article Type:	Protocol
Date Submitted by the Author:	13-Nov-2020
Complete List of Authors:	Cabecinha, Melissa; University College London, Research Department of Primary Care and Population Health<br>Solomon, Danielle; University College London, Institute for Global Health<br>Rait, Greta; University College London, Primary Care<br>Saunders, John; National Institute for Health Research , Health Protection Research Unit in Blood Borne and Sexually Transmitted Infections at University College London; University College London, Institute of Global Health, Research Department of Infection and Population Health<br>Mohammed, Hamish; Public Health England, HIV & STI Department<br>McDonagh, Lorraine; University College London, Research Department of Primary Care and Population Health
**Primary Subject Heading**:	HIV/AIDS
Secondary Subject Heading:	Sexual health
Keywords:	HIV & AIDS < INFECTIOUS DISEASES, PREVENTIVE MEDICINE, SEXUAL MEDICINE

SCHOLARONE™
Manuscripts

I, the Submitting Author has the right to grant and does grant on behalf of all authors of the Work (as defined in the below author licence), an exclusive licence and/or a non-exclusive licence for contributions from authors who are: i) UK Crown employees; ii) where BMJ has agreed a CC-BY licence shall apply, and/or iii) in accordance with the terms applicable for US Federal Government officers or employees acting as part of their official duties; on a worldwide, perpetual, irrevocable, royalty-free basis to BMJ Publishing Group Ltd ("BMJ") its licensees and where the relevant Journal is co-owned by BMJ to the co-owners of the Journal, to publish the Work in this journal and any other BMJ products and to exploit all rights, as set out in our licence.

The Submitting Author accepts and understands that any supply made under these terms is made by BMJ to the Submitting Author unless you are acting as an employee on behalf of your employer or a postgraduate student of an affiliated institution which is paying any applicable article publishing charge ("APC") for Open Access articles. Where the Submitting Author wishes to make the Work available on an Open Access basis (and intends to pay the relevant APC), the terms of reuse of such Open Access shall be governed by a Creative Commons licence – details of these licences and which Creative Commons licence will apply to this Work are set out in our licence referred to above.

Other than as permitted in any relevant BMJ Author's Self Archiving Policies, I confirm this Work has not been accepted for publication elsewhere, is not being considered for publication elsewhere and does not duplicate material already published. I confirm all authors consent to publication of this Work and authorise the granting of this licence.

For peer review only

EQUITY CONSIDERATIONS IN OUTCOME MEASURES OF THE HIV PRE-EXPOSURE PROPHYLAXIS

CARE CONTINUUM IN HIGH INCOME COUNTRIES: A SYSTEMATIC REVIEW PROTOCOL

Melissa Cabecinha[1]

(m.cabecinha@ucl.ac.uk)

Danielle Solomon[2]

(danielle.solomon@ucl.ac.uk)

Greta Rait[1,3]

(g.rait@ucl.ac.uk)

John Saunders[3,4]

(john.saunders@ucl.ac.uk)

Hamish Mohammed[5,6]

(hamish.mohammed@phe.gov.uk)

&

Lorraine K. McDonagh[1,3]

(l.mcdonagh@ucl.ac.uk)

[1]Research Department of Primary Care and Population Health, University College London,

[2]Institute for Global Health, University College London

[3]National Institute for Health Research Health Protection Research Unit in Blood Borne and Sexually Transmitted Infections at University College London

[4]UCL Centre for Clinical Research in Infection and Sexual Health, Institute for Global Health, University College London

For peer review only

[5]Blood Safety, Hepatitis, Sexually Transmitted Infections (STI) and HIV Division, National Infection Service, Public Health England

[6]Infection & Population Health, Institute for Global Health, University College, London

CORRESPONDENCE TO: Ms Melissa Cabecinha; m.cabecinha@@ucl.ac.uk; Research Department of Primary Care and Population Health, Upper third floor, UCL Medical School (Royal Free Campus), Rowland Kill Street, London, NW3 2PF

For peer review only

KEYWORDS: HIV Pre-Exposure Prophylaxis; HIV prevention; health service evaluation; health equity

WORD COUNT: 3115words

ABSTRACT

Introduction

HIV Pre-exposure prophylaxis (PrEP) is an effective intervention to reduce acquisition of HIV. PrEP provision has increased in recent years, however, it is not known whether PrEP implementation has been equitably implemented across all risk groups, particularly groups experiencing high levels of health inequity. A PrEP care-continuum (PCC) has been proposed to evaluate the success of PrEP implementation programmes, but the extent to which health equity characteristics are currently taken into account in the PCC has not been described. The objectives of this proposed systematic review are to 1) identify and collate outcome measure definitions for the main stages of the PCC (awareness, acceptability, uptake, adherence, and retention), 2) describe how equity characteristics are considered in outcome definitions of the PCC, and 3) describe data sources for capturing equity characteristics.

Methods and analysis

Quantitative studies published after 2012 will be included. Five databases (MEDLINE, PubMed, Embase, CINHAL, ASSIA) will be searched to identify English language publications that include an outcome measure definition of at least one of the five main stages of the PCC. Risk of bias will be assessed using the Effective Public Health Practice Project Quality Assessment Tool for Quantitative Studies. Data on outcome measure definitions and equity characteristics will be extracted. Results will be presented in a narrative synthesis and all findings will be reported in accordance with the Preferred Reporting Items for Systematic Reviews and Meta-Analyses guidelines.

Ethics and dissemination

Ethical approval is not required. The results will be disseminated via submission for publication to a peer-reviewed journal when complete. The review findings will have relevance to healthcare professionals, policymakers and commissioners in informing how to best evaluate PrEP implementation programmes and inform new implementation strategies for vulnerable and less advantaged populations.

For peer review only

ARTICLE SUMMARY

Strengths and limitations of this study

- This systematic review will be the first to collate outcome measures for the HIV Pre-Exposure Prophylaxis (PrEP) Care Continuum from real-world settings and investigate how equity characteristics are considered within existing measures.
- The findings from this review will help inform evaluation and monitoring of PrEP programmes and how to view these evaluations through an equity lens.
- This protocol has been written in accordance with published PRISMA-P guidelines, and the review will be reported in accordance with the PRISMA statement guidelines and the PRISMA-E 2012 equity extension.
- This review will not provide specific information on the effectiveness of existing PrEP programmes at reducing health inequity or population vulnerability.

For peer review only - http://bmjopen.bmj.com/site/about/guidelines.xhtml

For peer review only

INTRODUCTION

Oral HIV Pre-Exposure prophylaxis (HIV PrEP, hereafter referred to as PrEP) is a highly effective biomedical intervention for reducing HIV acquisition, and is recommended by the World Health Organisation (WHO) as part of a comprehensive approach to HIV prevention[1]. The efficacy of PrEP has been demonstrated in clinical trials[2,3], and PrEP provision has been increasing globally in recent years[4]. However, questions remain as to whether real-world implementation strategies are reaching some high-risk and vulnerable populations[5], particularly those populations experiencing high levels of health inequity.

Health Inequity and HIV

Health inequities are disparities in health status or access to healthcare that are considered unnecessary, avoidable, unfair and unjust[6]. Health equity is an important consideration in HIV prevention, as HIV disproportionately affects marginalised populations. Gender and sexual minorities[7–9], ethnic minorities[7,10], vulnerable populations such as people who inject drugs[11], people experiencing housing instability[12] and other social disadvantages[13] are at a greater risk of HIV acquisition compared to the general population. These categories are not mutually exclusive; an individual may identify with multiple categories concurrently[14] and the experience of belonging to multiple marginalized groups may contribute to an increased risk of acquiring HIV[15]. HIV prevention programmes, including PrEP provision programmes, must address the needs of marginalised populations and communities in order to have a sustained impact on the decline of HIV transmission[11].

Measureable Outcomes for PrEP Implementation

For peer review only - http://bmjopen.bmj.com/site/about/guidelines.xhtml

A PrEP Care Continuum (PCC) has been proposed[16,17] to evaluate the implementation of PrEP. The main stages of the PCC are as follows: awareness (knowledge of PrEP and indication of relevance), acceptability (whether an individual views PrEP as an acceptable tool for prevention), uptake (engagement in PrEP related care and initiation of PrEP), and adherence (retention in PrEP related care and adherence to treatment). Although the PCC provides a useful framework, in practice the outcomes have been reported with varying definitions. For example, in evaluating the "uptake" stage of the continuum, a study of veterans in the United States reports uptake as the absolute number of people initiating on PrEP within a given time-frame[18], whereas the South Africa Program defines uptake within a given programme as the proportion initiating PrEP after both a negative test for HIV and an offer of oral PrEP[19]. In England, the PrEP Impact Trial defines uptake as the proportion initiating oral PrEP among those meeting the eligibility criteria in participating sexual health clinics[20]. A common definition of uptake has been proposed for monitoring PrEP delivery to adolescent girls and young women in sub-Saharan Africa ("the number who initiate oral PrEP among those offered PrEP")[21] and consistent definitions for the outcome measures of the PCC would be beneficial for comparing the relative success of PrEP provision programmes.

Health Inequity and PrEP

These outcome measures are important for evaluating the overall success of PrEP programmes, identifying populations with low engagement, and ensuring that programmes do not increase health inequity among at-risk populations. The latter can be achieved, in part, by considering health equity characteristics when evaluating PrEP provision programmes. Structural and environmental factors that contribute to discrepancies in HIV rates have been shown to affect HIV treatment uptake and adherence[22,23], uptake of HIV prevention strategies in general[23] and for PrEP specifically. For example, lower levels of education, anticipated stigma, and higher PrEP cost have been associated with lower willingness to take PrEP[24]. Low adherence to PrEP has been associated with competing survival needs (e.g. financial instability, housing) [25] and substance use[26]. It is likely that other structural, social and environmental factors influence engagement at each stage of the PCC, such as those summarised by the PROGRESS-Plus framework[27,28].

The PROGRESS-Plus framework describes a sample of characteristics that drive disparities in health[28,29]. The acronym PROGRESS refers to Place of residence (e.g., rural/urban/inner city), Race/ethnicity/culture/language, Occupation, Gender/sex, Religion, Education, Socio-economic status, and Social capital. The "Plus" suffix includes additional categories that can attract discrimination for consideration: personal characteristics (such as age or having a disability), features of a relationship (such as being excluded from school or having unpaid care responsibilities), and time dependent relationships (such as having recently been hospitalized or receiving respite care).

Currently, the extent to which characteristics influencing health equity, such as those in the PROGRESS-Plus framework, are being investigated in outcome measures of the PCC is not well characterised. The evaluation of health equity within PrEP implementation

For peer review only - http://bmjopen.bmj.com/site/about/guidelines.xhtml

programmes could be improved by collating current approaches for evaluating outcome measures of the PCC and existing approaches to considering health equity within them.

In order to minimise heterogeneity, this review will focus on outcome measures of the PCC in high-income countries. Although factors influencing health equity in high-income countries are likely to be similar to those in low- and middle-income countries, how PrEP programmes are monitored, and the way vulnerable populations experience health inequity, may differ between these settings.

Aim

This paper is a protocol for a systematic review that aims to identify and collate definitions for outcome measures of the PCC and describe how, and the extent to which, characteristics that influence health equity are taken into account in these measures in high income countries.

Objectives

The objectives of this proposed systematic review are:

1) To identify and collate definitions of outcome measures used to evaluate the main stages of the PCC (awareness, acceptability, uptake, adherence and retention) for real-world settings in high income countries.

2) To explore whether and how studies reporting outcome measures of the PCC consider the effects of health equity characteristics when reporting PrEP awareness, acceptability, uptake, adherence and retention.

3) To describe the sources of data and methods used for capturing equity characteristics (e.g., self-reported survey data, administrative data, linked datasets)

This review aims to describe how outcome measures for the PCC are defined and to describe how health equity characteristics are currently taken into account for these measures. This review is not intended to summarise the effectiveness of PrEP programmes at reducing health inequity or population vulnerability.

METHOD DESIGN

Patient and public involvement

This protocol was informed by input from a Patient and Public Involvement (PPI) representative. A one-off PPI session was held to discuss the acceptability and accessibility of investigating equity considerations in the PCC and the appropriateness of using the PROGRESS-Plus framework to do so.

Protocol and registration

This protocol was reported according to the Preferred Reporting Items for Systematic Review and Meta-Analysis Protocols (PRIMSA-P) 2015 statement[30] (appendix 1) and has been registered with the PROSPERO international prospective register of systematic reviews

For peer review only - http://bmjopen.bmj.com/site/about/guidelines.xhtml

(Prospero registration number: CRD42020169779, available at: https://www.crd.york.ac.uk/prospero/display_record.php?RecordID=169779).

The review will be conducted and reported in accordance with the PRISMA statement guidelines[31] and the PRISMA-E 2012 equity extension[32].

Eligibility criteria

To be included in the review, papers will have to meet the following Population, Intervention, Context, Outcomes and Study design elements:

1. Population

Inclusion criteria: People resident in high-income countries with a national PrEP provision programme (i.e., where the national governing body has approved PrEP for use and it is available from health care facilities and/or bespoke initiatives) or demonstration/implementation project, regardless of sexual orientation/identity, age, gender identity, or occupation.

Exclusion criteria: People resident in countries without a national PrEP provision program or demonstration/implementation project; people resident in low- or middle-income countries.

2. Intervention

The issue to be reviewed is outcome measures for five stages of the PCC (awareness, acceptability, uptake, adherence and retention). Definitions of outcome measures for the PCC in real-world settings will be collated and reviewed, rather than the reported outcomes. For example, in studies reporting PrEP awareness, the definition of awareness and the metrics used to determine awareness are relevant to this review, whereas the reported level of awareness is not. As such, studies which include descriptions of how the main stages of the PCC will be evaluated but do not report these outcomes (e.g. study protocols) will be included.

Inclusion criteria: Studies reporting an outcome measure definition for at least one stage of the PCC in a real-world setting.

Exclusion Criteria: Studies that do not report an outcome measure definition for the PCC, randomised controlled trials (RCTs).

3. Context

Inclusion criteria: Studies conducted in high income countries, as defined by the World Bank country classifications[33]. Inequity is a relative concept, and inequities experienced by vulnerable populations in lower and middle-income countries may differ from that experienced by vulnerable populations in high-income countries with well-resourced healthcare settings.

Exclusion criteria: Studies conducted in low- or middle- income countries.

For peer review only - http://bmjopen.bmj.com/site/about/guidelines.xhtml

4. Outcomes

Primary outcomes: Studies will be included if they report at least one outcome measure definition for the PCC relating to PrEP provision in a real-world setting, i.e. an implementation trial, demonstration project or national programme. Studies only reporting absolute numbers for stages of the PCC will not be included.

Secondary outcomes: Data on whether factors influencing health equity (defined by the PROGRESS-Plus framework) are considered for the description of baseline characteristics of participants and/or when reporting measurable outcomes for the PCC.

5. Study Design

Inclusion criteria: Quantitative research studies including non-experimental observational studies (e.g. cohort, cross-sectional, and longitudinal studies) and abstracts from key international HIV/STI conferences (e.g. HIV/STI World Congress, International AIDS society, Conference on Retroviruses and Opportunistic Infections). Mixed method studies will be included providing a quantified outcome measure of the PCC is reported. Studies resulting from demonstration and/or implementation trials will also be included. Study protocols, policy frameworks and clinical guidelines will be included if they include definitions of outcome measures in relation to a real-world programme; for example definitions for how the numerator (e.g. the number of people with high adherence to PrEP) and denominator (e.g. the number of people taking PrEP) will be defined in a measure of PrEP adherence.

Exclusion Criteria: Qualitative studies, , review articles, case studies, studies not involving oral emtricitabine/tenofovir disoproxil fumarate as PrEP, and commentary or opinion pieces that do not contain a definition for an outcome measure of the PCC. Randomised controlled trials (RCTs) will also be excluded, as RCTs in high income countries predominately focus on safety and efficacy, rather than outcome measures of the PCC.

Information sources

Electronic searches

To identify studies, the following electronic databases will be searched: MEDLINE (via Ovid), EMBASE (via Ovid), PubMed, Cumulative Index to Nursing and Allied Health Literature (CINAHL) (via EBSCO Host), and Applied Social Sciences Index and Abstracts (ASSIA) (via ProQuest).

Search strategy

Restrictions on English language articles as well as for articles published between 2012 (the year the United States Federal Drug Administration approved Truvada [emtricitabine/tenofovir disoproxil] fumarate for PrEP use[34]) and the present will be applied.

Medical Subject headings (MeSH), subject headings and keyword lists will be created by using language that describes 1) oral PrEP and 2) the following outcome measures of the PCC: awareness, acceptability, uptake, adherence and retention. Boolean combinations will

For peer review only - http://bmjopen.bmj.com/site/about/guidelines.xhtml

be employed to refine the search strategy. Several key articles will be identified in the preliminary searches and used to test the sensitivity of the search strategy.

The search strategy that will be used to search MEDLINE (via the Ovid platform) is presented in appendix 2. Search terms will be modified for databases where the indexing of subject headings differs from the terms used in MEDLINE.

The references of articles meeting the inclusion criteria will be searched to identify any relevant papers missed in the electronic searches.

Study selection

All citations retrieved from the electronic searches will be imported into an EndNote database. After duplicates are removed, the remaining studies will be screened by title and abstract by one reviewer, with a random sample independently reviewed by a second reviewer. Reviewers will compare their decisions throughout the process and discuss all references where there was a disagreement. A third reviewer will be asked to adjudicate where discrepancies cannot be resolved. Studies remaining after the initial title and abstract screen will undergo full text screening. Following the full-text screening, data will be extracted from all studies that meet the inclusion criteria. A flow diagram will be produced to show selection process, according to PRISMA guidelines[35], with explanation for those excluded at each stage.

Data extraction and management

Data will be extracted into a pilot-tested, standardised form by one reviewer with a random sample checked by a second reviewer. Any discrepancies will be resolved by discussion or adjudication by a third reviewer where necessary.

All studies that meet the inclusion criteria will be described in terms of:

- Bibliographic information (first author, year of study, country of study)
- Study aims and design (including study population, sample size, and data collection methods)
- Participants, demographic characteristics and number of baseline PROGRESS-Plus equity characteristics reported
- Outcome measure definitions (for PrEP awareness, acceptability, uptake, adherence, and retention).
- Whether the study considered PROGRESS-Plus equity characteristics when measuring outcomes of the PCC.

For studies that consider PROGRESS-Plus equity characteristics in outcome measures, further data extraction detailing how the PROGRESS-Plus characteristics are expressed, and additional information on the study methods and study results will be extracted into an expanded data collection form.

Risk of bias assessment of included studies

For peer review only - http://bmjopen.bmj.com/site/about/guidelines.xhtml

The quality of each paper included in the data extraction stage will be assessed independently by two reviewers, using the Effective Public Health Practice Project (EPHPP) "Quality Assessment Tool for Quantitative Studies"[36]. Individual studies will be categorised as "strong", "moderate", or "weak" in the following domains: selection bias, study design, confounders, blinding, data collection method, and withdrawals and drop outs. Studies will also be given a global score to indicate overall quality. Any discrepancies between the two reviewers will be resolved by consensus, or by a third reviewer if necessary.

Data synthesis and analysis

A narrative synthesis of results will be conducted. Individual study characteristics and outcomes will be summarised and presented in an evidence table.

To assess the extent to which studies consider the effects of PROGRESS-Plus equity characteristics in the outcome measures of the PCC, a two-stage analysis will be carried out. At the first stage, data will be presented on PCC outcome measure definitions and whether the study considers any of the PROGRESS-Plus equity characteristics for the description of baseline characteristics of study participants. The second stage we will describe whether the study considered PROGRESS-Plus characteristics in outcome measures of the PrEP continuum, and which methods were used (e.g. consideration as confounders by adjusting in multivariable analysis, as effect modifiers in stratified analysis, using interaction terms in multivariable analysis etc.). The ways in which inequities are expressed (i.e. whether absolute or relative) will also be reported, as the decision about whether to measure inequity in relative or absolute terms may influence their interpretation.

DISCUSSION

To the best of the author's knowledge, this is the first systematic review to collate definitions for outcome measures of the PCC and describe the extent to (and ways in which) equity characteristics are considered in these outcomes. The findings will have relevance to healthcare professionals, policymakers and commissioners in informing how to evaluate PrEP implementation programmes, and how to view these evaluations through an equity lens to optimise HIV prevention strategies and inform new implementation strategies for vulnerable and less advantaged populations.

Ethics and Dissemination

Ethical approval is not required for this study. When complete, the review results will be submitted for publication in a peer-reviewed journal and submitted to be presented at national and international conferences (where eligible). The findings, dissemination strategy, and lay summary will be discussed with a PPI panel.

Authors contributions

MC and LMD designed the study. MC developed and refined the study protocol with contributions from all co-authors (DS, JS, GR, HM and LMD). MC prepared the manuscript. MC will undertake data collection (literature search, data extraction), analysis,

For peer review only - http://bmjopen.bmj.com/site/about/guidelines.xhtml

interpretation and report writing. All co-investigators will contribute to the design, analysis, interpretation and report writing. All authors read and approved the final manuscript. The authors would like to thank the PPI representative for their contributions to the research question and study design.

Funding Statement

MC is funded by the Medical Research Council Doctoral Training Partnership (grant number: MR/N013867/1). The research is funded by the National Institute for Health Research Health Protection Research Unit (NIHR HPRU) in Blood Borne and Sexually Transmitted Infections at University College London in partnership with Public Health England (reference number: NIHR200911). The views expressed in this publication are those of the authors and not necessarily those of the NHS, the Medical Research Council, the National Institute for Health Research, the Department of Health or Public Health England.

Competing interests statement:

None declared

REFERENCES

1. World Health Organization. *Guideline on When To Start Antiretroviral Therapy and on Pre-Exposure Prophylaxis for HIV*. (2015).

2. Grant, R. M. *et al.* Preexposure Chemoprophylaxis for HIV Prevention in Men Who Have Sex with Men. *N. Engl. J. Med.* **363**, 2587–2599 (2010).

3. Baeten, J. M. *et al.* Antiretroviral Prophylaxis for HIV Prevention in Heterosexual Men and Women. *N. Engl. J. Med.* **367**, 399–410 (2012).

4. Country Updates – PrEPWatch. https://www.prepwatch.org/in-practice/country-updates/.

5. Pyra, M. N. *et al.* Global implementation of PrEP for HIV prevention: setting expectations for impact. *J. Int. AIDS Soc.* **22**, (2019).

6. Marmot, M., Friel, S., Bell, R., Houweling, T. A. & Taylor, S. Closing the gap in a generation: health equity through action on the social determinants of health. *Lancet* **372**, 1661–1669 (2008).

7. Public Health England. Trends in new HIV diagnoses and people receiving HIV-related care in the United Kingdom: data to the end of December 2017. *Heal. Prot. Rep. Adv. Access Rep. [v2]* **volume 12**, (2018).

8. Centers for Disease Control and Prevention. HIV and Young Men Who Have Sex With Men. *Natl. Cent. HIV/AIDS, VIral Hepatitis, STD TB Prev.* 1–4 (2014).

9. Mayer, K. H., Grinsztejn, B. & El-Sadr, W. M. Transgender People and HIV Prevention. *JAIDS J. Acquir. Immune Defic. Syndr.* **72**, S207–S209 (2016).

10. Grossman, C. I., Purcell, D. W., Rotheram-Borus, M. J. & Veniegas, R. Opportunities for HIV

For peer review only - http://bmjopen.bmj.com/site/about/guidelines.xhtml

combination prevention to reduce racial and ethnic health disparities. *Am. Psychol.* **68**, 237–246 (2013).

11. UNAIDS. *Miles to go: closing gaps, breaking barriers, fighting injustices*. (2018).

12. Elifson, K. W., Sterk, C. E. & Theall, K. P. Safe living: The impact of unstable housing conditions on HIV risk reduction among female drug users. *AIDS Behav.* **11**, (2007).

13. Pellowski, J. A., Kalichman, S. C., Matthews, K. A. & Adler, N. A pandemic of the poor: social disadvantage and the U.S. HIV epidemic. *Am. Psychol.* **68**, 197–209.

14. Rapid Response Service. *Intersectionality in HIV and Other Health-Related Research*. www.health.gov.on.ca/en/public/ (2013).

15. Collins, P. Y., von Unger, H. & Armbrister, A. Church ladies, good girls, and locas: Stigma and the intersection of gender, ethnicity, mental illness, and sexuality in relation to HIV risk. *Soc. Sci. Med.* **67**, 389–397 (2008).

16. Kelley, C. F. *et al.* Applying a PrEP Continuum of Care for Men Who Have Sex with Men in Atlanta, Georgia. *Clin. Infect. Dis.* **61**, 1590–1597 (2015).

17. Nunn, A. S. *et al.* Defining the HIV pre-exposure prophylaxis care continuum. *AIDS* **31**, 731–734 (2017).

18. Chartier, M. *et al.* Accessibility and Uptake of Pre-Exposure Prophylaxis for HIV Prevention in the Veterans Health Administration. *Fed Pr.* **35**, S42-s48 (2018).

19. South African National Department of Health (NDoH). Pre-exposure prophylaxis implementation in South Africa. in *Presented at the 19th Annual International Conference on AIDS and STIs in Africa (ICASA)* (2017).

20. PrEP Impact Trial. A pragmatic health technology assessment of PrEP and implementation. Trial Protocol. v3.0. https://www.prepimpacttrial.org.uk/protocol.

21. Dunbar, M. S. *et al.* Understanding and measuring uptake and coverage of oral pre-exposure prophylaxis delivery among adolescent girls and young women in sub-Saharan Africa. *Sexual Health* vol. 15 513–521 (2018).

22. Duff, P. *et al.* Barriers to Viral Suppression among Female Sex Workers: Role of Structural and Intimate Partner Dynamics. *J. Acquir. Immune Defic. Syndr.* **73**, 83–90 (2016).

23. Gari, S. *et al.* Access to HIV/AIDS care: A systematic review of socio-cultural determinants in low and high income countries. *BMC Health Services Research* vol. 13 (2013).

24. Yi, S. *et al.* Awareness and willingness to use HIV pre-exposure prophylaxis among men who have sex with men in low- and middle-income countries: A systematic review and meta-analysis: A. *Journal of the International AIDS Society* vol. 20 (2017).

25. Janet, J. M. *et al.* Adherence to PrEP among young men who have sex with men participating in a sexual health services demonstration project in Alameda County, California. *J Acquir Immune Defic Syndr* (2019) doi:10.1097/qai.0000000000002051.

26. Zucker, J. *et al.* Predictors of Disengagement in Care for Individuals Receiving Pre-Exposure Prophylaxis (PrEP). *J Acquir Immune Defic Syndr* (2019) doi:10.1097/qai.0000000000002054.

27. O'Neill, J. *et al.* Applying an equity lens to interventions: Using PROGRESS ensures consideration of socially stratifying factors to illuminate inequities in health. *J. Clin. Epidemiol.* **67**, 56–64 (2014).

For peer review only - http://bmjopen.bmj.com/site/about/guidelines.xhtml

28. Kavanagh, J., Oliver, S. & Lorenc, T. Reflections on developing and using PROGRESS-Plus. *Equity Updat.* **2**, 1–3 (2008).

29. Evans, T. & Brown, H. Road traffic crashes: operationalizing equity in the context of health sector reform. *Inj. Control Saf. Promot.* **10**, 11–2.

30. Moher, D. *et al.* Preferred reporting items for systematic review and meta-analysis protocols (PRISMA-P) 2015 statement. *Rev. Esp. Nutr. Humana y Diet.* **20**, 148–160 (2016).

31. Liberati, A. *et al.* The PRISMA statement for reporting systematic reviews and meta-analyses of studies that evaluate healthcare interventions: explanation and elaboration. *BMJ* **339**, (2009).

32. Welch, V. *et al.* PRISMA-Equity 2012 Extension: Reporting Guidelines for Systematic Reviews with a Focus on Health Equity. *PLoS Med.* **9**, (2012).

33. WorldBank. World Bank Country and Lending Groups – World Bank Data Help Desk. https://datahelpdesk.worldbank.org/knowledgebase/articles/906519 (2019).

34. FDA. Truvada for PrEP Fact Sheet : Ensuring Safe and Proper Use. 1–2 (2012).

35. Shamseer, L. *et al.* Preferred reporting items for systematic review and meta-analysis protocols (prisma-p) 2015: Elaboration and explanation. *BMJ (Online)* vol. 350 (2015).

36. Quality Assessment Tool for Quantitative Studies. https://www.ephpp.ca/quality-assessment-tool-for-quantitative-studies/.

For peer review only

For peer review only - http://bmjopen.bmj.com/site/about/guidelines.xhtml

PRISMA-P (Preferred Reporting Items for Systematic review and Meta-Analysis Protocols) 2015 checklist: recommended items to address in a systematic review protocol*

Section and topic	Item No	Checklist item
**ADMINISTRATIVE INFORMATION**		
Title:		
Identification	1a	Identify the report as a protocol of a systematic review
Update	1b	If the protocol is for an update of a previous systematic review, identify as such
Registration	2	If registered, provide the name of the registry (such as PROSPERO) and registration number
Authors:		
Contact	3a	Provide name, institutional affiliation, e-mail address of all protocol authors; provide physical mailing address of corresponding author
Contributions	3b	Describe contributions of protocol authors and identify the guarantor of the review
Amendments	4	If the protocol represents an amendment of a previously completed or published protocol, identify as such and list changes; otherwise, state plan for documenting important protocol amendments
Support:		
Sources	5a	Indicate sources of financial or other support for the review
Sponsor	5b	Provide name for the review funder and/or sponsor
Role of sponsor or funder	5c	Describe roles of funder(s), sponsor(s), and/or institution(s), if any, in developing the protocol
**INTRODUCTION**		
Rationale	6	Describe the rationale for the review in the context of what is already known
Objectives	7	Provide an explicit statement of the question(s) the review will address with reference to participants, interventions, comparators, and outcomes (PICO)
**METHODS**		
Eligibility criteria	8	Specify the study characteristics (such as PICO, study design, setting, time frame) and report characteristics (such as years considered, language, publication status) to be used as criteria for eligibility for the review
Information sources	9	Describe all intended information sources (such as electronic databases, contact with study authors, trial registers or other grey literature sources) with planned dates of coverage
Search strategy	10	Present draft of search strategy to be used for at least one electronic database, including planned limits, such that it could be repeated
Study records:		
Data management	11a	Describe the mechanism(s) that will be used to manage records and data throughout the review

For peer review only

Selection process	11b	State the process that will be used for selecting studies (such as two independent reviewers) through each phase of the review (that is, screening, eligibility and inclusion in meta-analysis)
Data collection process	11c	Describe planned method of extracting data from reports (such as piloting forms, done independently, in duplicate), any processes for obtaining and confirming data from investigators
Data items	12	List and define all variables for which data will be sought (such as PICO items, funding sources), any pre-planned data assumptions and simplifications
Outcomes and prioritization	13	List and define all outcomes for which data will be sought, including prioritization of main and additional outcomes, with rationale
Risk of bias in individual studies	14	Describe anticipated methods for assessing risk of bias of individual studies, including whether this will be done at the outcome or study level, or both; state how this information will be used in data synthesis
Data synthesis	15a	Describe criteria under which study data will be quantitatively synthesised
	15b	If data are appropriate for quantitative synthesis, describe planned summary measures, methods of handling data and methods of combining data from studies, including any planned exploration of consistency (such as I^2, Kendall's τ)
	15c	Describe any proposed additional analyses (such as sensitivity or subgroup analyses, meta-regression)
	15d	If quantitative synthesis is not appropriate, describe the type of summary planned
Meta-bias(es)	16	Specify any planned assessment of meta-bias(es) (such as publication bias across studies, selective reporting within studies)
Confidence in cumulative evidence	17	Describe how the strength of the body of evidence will be assessed (such as GRADE)

*** It is strongly recommended that this checklist be read in conjunction with the PRISMA-P Explanation and Elaboration (cite when available) for important clarification on the items. Amendments to a review protocol should be tracked and dated. The copyright for PRISMA-P (including checklist) is held by the PRISMA-P Group and is distributed under a Creative Commons Attribution Licence 4.0.**

From: Shamseer L, Moher D, Clarke M, Ghersi D, Liberati A, Petticrew M, Shekelle P, Stewart L, PRISMA-P Group. Preferred reporting items for systematic review and meta-analysis protocols (PRISMA-P) 2015: elaboration and explanation. BMJ. 2015 Jan 2;349(jan02 1):g7647.

For peer review only

Appendix 2

Systematic Review Search Strategy

1. exp HIV Infections/
2. Pre-Exposure Prophylaxis/
3. Chemoprevention/
4. Anti-HIV Agents/
5. Anti-Retroviral Agents/
6. 1 or 4 or 5
7. 2 or 3
8. 6 and 7
9. ((pre-exposure or preexposure or pre exposure) adj2 prophyla*).tw
10. PrEP.tw
11. ((pre-exposure or preexposure) adj2 chemoprophyla*).tw
12. 10 or 11
13. chemoprophylaxis.tw
14. chemo-prophylaxis.tw
15. chemoprevention.tw
16. chemo-prevention.tw
17. 12 or 13 or 14 or 15 or 16
18. truvada.tw
19. Emtricitabine/
20. Tenofovir/
21. 19 and 20
22. 18 or 21
23. (HIV or Human Immunodeficiency virus).tw
24. 17 and 23
25. 8 or 9 or 22 or 24
26. limit 25 to yr="2012 -Current"
27. limit 26 to (english language and humans)
28. exp Attitude to Health/
29. exp "Treatment Adherence and Compliance"/

For peer review only

30. Program Evaluation/mt, st, sn [Methods, Standards, Statistics & Numerical Data]

31. ((care or care-) adj2 (cascade or continuum)).tw

32. ((prevention or prevention-) adj2 (cascade or continuum)).tw

33. (non-adherence or nonadherence or non adherence).tw

34. (noncompliance or non-compliance or non compliance).tw

35. access.tw

36. adhere*.tw

37. attitude*.tw

38. aware*.tw

39. compliance.tw

40. compliant.tw

41. demand.tw

42. disengage*.tw

43. enable*.tw

44. engage*.tw

45. evaluat*.tw

46. experience*.tw

47. implement*.tw

48. interest.tw

49. knowledge.tw

50. measure*.tw

51. perception*.tw

52. retain*.tw

53. retention.tw

54. uptake.tw

55. 28 or 29 or 30 or 31 or 32 or 33 or 34 or 35 or 36 or 37 or 38 or 39 or 40 or 41 or 42 or 43 or 44 or 45 or 46 or 47 or 48 or 49 or 50 or 51 or 52 or 53 or 54

56. 27 and 55

For peer review only

Reporting checklist for protocol of a systematic review.

Based on the PRISMA-P guidelines.

Instructions to authors

Complete this checklist by entering the page numbers from your manuscript where readers will find each of the items listed below.

Your article may not currently address all the items on the checklist. Please modify your text to include the missing information. If you are certain that an item does not apply, please write "n/a" and provide a short explanation.

Upload your completed checklist as an extra file when you submit to a journal.

In your methods section, say that you used the PRISMA-Preporting guidelines, and cite them as:

Moher D, Shamseer L, Clarke M, Ghersi D, Liberati A, Petticrew M, Shekelle P, Stewart LA. Preferred Reporting Items for Systematic Review and Meta-Analysis Protocols (PRISMA-P) 2015 statement. Syst Rev. 2015;4(1):1.

		Reporting Item	Page Number
**Title**			
Identification	#1a	Identify the report as a protocol of a systematic review	pg 1, line 2
Update	#1b	If the protocol is for an update of a previous systematic review, identify as such	N/A
**Registration**			
	#2	If registered, provide the name of the registry (such as PROSPERO) and registration number	pg 6, line 200 - 201
**Authors**			
Contact	#3a	Provide name, institutional affiliation, e-mail address of all protocol authors; provide physical mailing address of corresponding author	pg1, line 4-26
Contribution	#3b	Describe contributions of protocol authors and identify the	pg10, line 347-

For peer review only

		guarantor of the review	353
**Amendments**			
	#4	If the protocol represents an amendment of a previously completed or published protocol, identify as such and list changes; otherwise, state plan for documenting important protocol amendments	N/A
**Support**			
Sources	#5a	Indicate sources of financial or other support for the review	pg 10, line 355 - 361
Sponsor	#5b	Provide name for the review funder and / or sponsor	N/A
Role of sponsor or funder	#5c	Describe roles of funder(s), sponsor(s), and / or institution(s), if any, in developing the protocol	N/A
**Introduction**			
Rationale	#6	Describe the rationale for the review in the context of what is already known	pg 4-5, line 103 -170
Objectives	#7	Provide an explicit statement of the question(s) the review will address with reference to participants, interventions, comparators, and outcomes (PICO)	pg. 5-6, line 178 - 189
**Methods**			
Eligibility criteria	#8	Specify the study characteristics (such as PICO, study design, setting, time frame) and report characteristics (such as years considered, language, publication status) to be used as criteria for eligibility for the review	pg. 6-7, line 207-250
Information sources	#9	Describe all intended information sources (such as electronic databases, contact with study authors, trial registers or other grey literature sources) with planned dates of coverage	pg. 8, line 261 - 280
Search strategy	#10	Present draft of search strategy to be used for at least one electronic database, including planned limits, such that it could be repeated	pg. 8, line 276 – 278(and appendix 2)
Study records - data management	#11a	Describe the mechanism(s) that will be used to manage records and data throughout the review	pg. 8, line 282 – 283

For peer review only

Study records - selection process	#11b	State the process that will be used for selecting studies (such as two independent reviewers) through each phase of the review (that is, screening, eligibility and inclusion in meta-analysis)	pg. 8-9, line 282 – 291
Study records - data collection process	#11c	Describe planned method of extracting data from reports (such as piloting forms, done independently, in duplicate), any processes for obtaining and confirming data from investigators	pg. 9, line 292 – 295
Data items	#12	List and define all variables for which data will be sought (such as PICO items, funding sources), any pre-planned data assumptions and simplifications	pg. 9, line 296 – 309
Outcomes and prioritization	#13	List and define all outcomes for which data will be sought, including prioritization of main and additional outcomes, with rationale	pg. 7, line 237 – 244
Risk of bias in individual studies	#14	Describe anticipated methods for assessing risk of bias of individual studies, including whether this will be done at the outcome or study level, or both; state how this information will be used in data synthesis	pg. 9, line 310 – 317
Data synthesis	#15a	Describe criteria under which study data will be quantitatively synthesised	N/A
Data synthesis	#15b	If data are appropriate for quantitative synthesis, describe planned summary measures, methods of handling data and methods of combining data from studies, including any planned exploration of consistency (such as I2, Kendall's τ)	N/A
Data synthesis	#15c	Describe any proposed additional analyses (such as sensitivity or subgroup analyses, meta-regression)	N/A
Data synthesis	#15d	If quantitative synthesis is not appropriate, describe the type of summary planned	pg. 9, line 318 – 331
Meta-bias(es)	#16	Specify any planned assessment of meta-bias(es) (such as publication bias across studies, selective reporting within studies)	N/A
Confidence in cumulative	#17	Describe how the strength of the body of evidence will be assessed (such as GRADE)	pg. 9, 310 – 317

For peer review only

evidence

The PRISMA-P checklist is distributed under the terms of the Creative Commons Attribution License CC-BY 4.0. This checklist was completed on 19. May 2020 using https://www.goodreports.org/, a tool made by the EQUATOR Network in collaboration with Penelope.ai

For peer review only

BMJ Open

Equity considerations in outcome measures of the HIV Pre-Exposure prophylaxis care continuum in high income countries: a systematic review protocol

Journal:	*BMJ Open*
Manuscript ID	bmjopen-2020-040701.R2
Article Type:	Protocol
Date Submitted by the Author:	20-Nov-2020
Complete List of Authors:	Cabecinha, Melissa; University College London, Research Department of Primary Care and Population Health<br>Solomon, Danielle; University College London, Institute for Global Health<br>Rait, Greta; University College London, Primary Care<br>Saunders, John; National Institute for Health Research , Health Protection Research Unit in Blood Borne and Sexually Transmitted Infections at University College London; University College London, Institute of Global Health, Research Department of Infection and Population Health<br>Mohammed, Hamish; Public Health England, HIV & STI Department<br>McDonagh, Lorraine; University College London, Research Department of Primary Care and Population Health
**Primary Subject Heading**:	HIV/AIDS
Secondary Subject Heading:	Sexual health
Keywords:	HIV & AIDS < INFECTIOUS DISEASES, PREVENTIVE MEDICINE, SEXUAL MEDICINE

SCHOLARONE™
Manuscripts

I, the Submitting Author has the right to grant and does grant on behalf of all authors of the Work (as defined in the below author licence), an exclusive licence and/or a non-exclusive licence for contributions from authors who are: i) UK Crown employees; ii) where BMJ has agreed a CC-BY licence shall apply, and/or iii) in accordance with the terms applicable for US Federal Government officers or employees acting as part of their official duties; on a worldwide, perpetual, irrevocable, royalty-free basis to BMJ Publishing Group Ltd ("BMJ") its licensees and where the relevant Journal is co-owned by BMJ to the co-owners of the Journal, to publish the Work in this journal and any other BMJ products and to exploit all rights, as set out in our licence.

The Submitting Author accepts and understands that any supply made under these terms is made by BMJ to the Submitting Author unless you are acting as an employee on behalf of your employer or a postgraduate student of an affiliated institution which is paying any applicable article publishing charge ("APC") for Open Access articles. Where the Submitting Author wishes to make the Work available on an Open Access basis (and intends to pay the relevant APC), the terms of reuse of such Open Access shall be governed by a Creative Commons licence – details of these licences and which Creative Commons licence will apply to this Work are set out in our licence referred to above.

Other than as permitted in any relevant BMJ Author's Self Archiving Policies, I confirm this Work has not been accepted for publication elsewhere, is not being considered for publication elsewhere and does not duplicate material already published. I confirm all authors consent to publication of this Work and authorise the granting of this licence.

For peer review only

EQUITY CONSIDERATIONS IN OUTCOME MEASURES OF THE HIV PRE-EXPOSURE PROPHYLAXIS CARE CONTINUUM IN HIGH INCOME COUNTRIES: A SYSTEMATIC REVIEW PROTOCOL

Melissa Cabecinha[1]

(m.cabecinha@ucl.ac.uk)

Danielle Solomon[2]

(danielle.solomon@ucl.ac.uk)

Greta Rait[1,3]

(g.rait@ucl.ac.uk)

John Saunders[3,4]

(john.saunders@ucl.ac.uk)

Hamish Mohammed[5,6]

(hamish.mohammed@phe.gov.uk)

&

Lorraine K. McDonagh[1,3]

(l.mcdonagh@ucl.ac.uk)

[1]Research Department of Primary Care and Population Health, University College London,

[2]Institute for Global Health, University College London

[3]National Institute for Health Research Health Protection Research Unit in Blood Borne and Sexually Transmitted Infections at University College London

[4]UCL Centre for Clinical Research in Infection and Sexual Health, Institute for Global Health, University College London

For peer review only

For peer review only - http://bmjopen.bmj.com/site/about/guidelines.xhtml

[5]Blood Safety, Hepatitis, Sexually Transmitted Infections (STI) and HIV Division, National Infection Service, Public Health England

[6]Infection & Population Health, Institute for Global Health, University College, London

CORRESPONDENCE TO: Ms Melissa Cabecinha; m.cabecinha@@ucl.ac.uk; Research Department of Primary Care and Population Health, Upper third floor, UCL Medical School (Royal Free Campus), Rowland Kill Street, London, NW3 2PF

KEYWORDS: HIV Pre-Exposure Prophylaxis; HIV prevention; health service evaluation; health equity

WORD COUNT: 3140 words

ABSTRACT

Introduction

HIV Pre-exposure prophylaxis (PrEP) is an effective intervention to reduce acquisition of HIV. PrEP provision has increased in recent years, however, it is not known whether PrEP implementation has been equitably implemented across all risk groups, particularly groups experiencing high levels of health inequity. A PrEP care-continuum (PCC) has been proposed to evaluate the success of PrEP implementation programmes, but the extent to which health equity characteristics are currently taken into account in the PCC has not been described. The objectives of this proposed systematic review are to 1) identify and collate outcome measure definitions for the main stages of the PCC (awareness, acceptability, uptake, adherence, and retention), 2) describe how equity characteristics are considered in outcome definitions of the PCC, and 3) describe data sources for capturing equity characteristics.

Methods and analysis

Quantitative studies published between January 1st, 2012 and March 3rd 2020 will be included. Five databases (MEDLINE, PubMed, Embase, CINHAL, ASSIA) will be searched to identify English language publications that include an outcome measure definition of at least one of the five main stages of the PCC. Risk of bias will be assessed using the Effective Public Health Practice Project Quality Assessment Tool for Quantitative Studies. Data on outcome measure definitions and equity characteristics will be extracted. Results will be presented in a narrative synthesis and all findings will be reported in accordance with the Preferred Reporting Items for Systematic Reviews and Meta-Analyses guidelines.

Ethics and dissemination

For peer review only

Ethical approval is not required. The results will be disseminated via submission for publication to a peer-reviewed journal when complete. The review findings will have relevance to healthcare professionals, policymakers and commissioners in informing how to best evaluate PrEP implementation programmes and inform new implementation strategies for vulnerable and less advantaged populations.

ARTICLE SUMMARY

Strengths and limitations of this study

- This systematic review will be the first to collate outcome measures for the HIV Pre-Exposure Prophylaxis (PrEP) Care Continuum from real-world settings and investigate how equity characteristics are considered within existing measures.

- The findings from this review will help inform evaluation and monitoring of PrEP programmes and how to view these evaluations through an equity lens.

- This protocol has been written in accordance with published PRISMA-P guidelines, and the review will be reported in accordance with the PRISMA statement guidelines and the PRISMA-E 2012 equity extension.

- This review will not provide specific information on the effectiveness of existing PrEP programmes at reducing health inequity or population vulnerability.

For peer review only - http://bmjopen.bmj.com/site/about/guidelines.xhtml

INTRODUCTION

Oral HIV Pre-Exposure prophylaxis (HIV PrEP, hereafter referred to as PrEP) is a highly effective biomedical intervention for reducing HIV acquisition, and is recommended by the World Health Organisation (WHO) as part of a comprehensive approach to HIV prevention[1]. The efficacy of PrEP has been demonstrated in clinical trials[2,3], and PrEP provision has been increasing globally in recent years[4]. However, questions remain as to whether real-world implementation strategies are reaching some high-risk and vulnerable populations[5], particularly those populations experiencing high levels of health inequity.

Health Inequity and HIV

Health inequities are disparities in health status or access to healthcare that are considered unnecessary, avoidable, unfair and unjust[6]. Health equity is an important consideration in HIV prevention, as HIV disproportionately affects marginalised populations. Gender and sexual minorities[7–9], ethnic minorities[7,10], vulnerable populations such as people who inject drugs[11], people experiencing housing instability[12] and other social disadvantages[13] are at a greater risk of HIV acquisition compared to the general population. These categories are not mutually exclusive; an individual may identify with multiple categories concurrently[14] and the experience of belonging to multiple marginalized groups may contribute to an increased risk of acquiring HIV[15]. HIV prevention programmes, including PrEP provision programmes, must address the needs of marginalised populations and communities in order to have a sustained impact on the decline of HIV transmission[11].

Measureable Outcomes for PrEP Implementation

For peer review only - http://bmjopen.bmj.com/site/about/guidelines.xhtml

A PrEP Care Continuum (PCC) has been proposed[16,17] to evaluate the implementation of PrEP. The main stages of the PCC are as follows: awareness (knowledge of PrEP and indication of relevance), acceptability (whether an individual views PrEP as an acceptable tool for prevention), uptake (engagement in PrEP related care and initiation of PrEP), and adherence (retention in PrEP related care and adherence to treatment). Although the PCC provides a useful framework, in practice the outcomes have been reported with varying definitions. For example, in evaluating the "uptake" stage of the continuum, a study of veterans in the United States reports uptake as the absolute number of people initiating on PrEP within a given time-frame[18], whereas the South Africa Program defines uptake within a given programme as the proportion initiating PrEP after both a negative test for HIV and an offer of oral PrEP[19]. In England, the PrEP Impact Trial defines uptake as the proportion initiating oral PrEP among those meeting the eligibility criteria in participating sexual health clinics[20]. A common definition of uptake has been proposed for monitoring PrEP delivery to adolescent girls and young women in sub-Saharan Africa ("the number who initiate oral PrEP among those offered PrEP")[21] and consistent definitions for the outcome measures of the PCC would be beneficial for comparing the relative success of PrEP provision programmes.

Health Inequity and PrEP

These outcome measures are important for evaluating the overall success of PrEP programmes, identifying populations with low engagement, and ensuring that programmes do not increase health inequity among at-risk populations. The latter can be achieved, in part, by considering health equity characteristics when evaluating PrEP provision programmes. Structural and environmental factors that contribute to discrepancies in HIV rates have been shown to affect HIV treatment uptake and adherence[22,23], uptake of HIV prevention strategies in general[23] and for PrEP specifically. For example, lower levels of education, anticipated stigma, and higher PrEP cost have been associated with lower willingness to take PrEP[24]. Low adherence to PrEP has been associated with competing survival needs (e.g. financial instability, housing) [25] and substance use[26]. It is likely that other structural, social and environmental factors influence engagement at each stage of the PCC, such as those summarised by the PROGRESS-Plus framework[27,28].

The PROGRESS-Plus framework describes a sample of characteristics that drive disparities in health[28,29]. The acronym PROGRESS refers to Place of residence (e.g., rural/urban/inner city), Race/ethnicity/culture/language, Occupation, Gender/sex, Religion, Education, Socio-economic status, and Social capital. The "Plus" suffix includes additional categories that can attract discrimination for consideration: personal characteristics (such as age or having a disability), features of a relationship (such as being excluded from school or having unpaid care responsibilities), and time dependent relationships (such as having recently been hospitalized or receiving respite care).

Currently, the extent to which characteristics influencing health equity, such as those in the PROGRESS-Plus framework, are being investigated in outcome measures of the PCC is not well characterised. The evaluation of health equity within PrEP implementation

For peer review only - http://bmjopen.bmj.com/site/about/guidelines.xhtml

programmes could be improved by collating current approaches for evaluating outcome measures of the PCC and existing approaches to considering health equity within them.

In order to minimise heterogeneity, this review will focus on outcome measures of the PCC in high-income countries. Although factors influencing health equity in high-income countries are likely to be similar to those in low- and middle-income countries, how PrEP programmes are monitored, and the way vulnerable populations experience health inequity, may differ between these settings.

Aim

This paper is a protocol for a systematic review that aims to identify and collate definitions for outcome measures of the PCC and describe how, and the extent to which, characteristics that influence health equity are taken into account in these measures in high income countries.

Objectives

The objectives of this proposed systematic review are:

1) To identify and collate definitions of outcome measures used to evaluate the main stages of the PCC (awareness, acceptability, uptake, adherence and retention) for real-world settings in high income countries.

2) To explore whether and how studies reporting outcome measures of the PCC consider the effects of health equity characteristics when reporting PrEP awareness, acceptability, uptake, adherence and retention.

3) To describe the sources of data and methods used for capturing equity characteristics (e.g., self-reported survey data, administrative data, linked datasets)

This review aims to describe how outcome measures for the PCC are defined and to describe how health equity characteristics are currently taken into account for these measures. This review is not intended to summarise the effectiveness of PrEP programmes at reducing health inequity or population vulnerability.

METHOD DESIGN

Patient and public involvement

This protocol was informed by input from a Patient and Public Involvement (PPI) representative. A one-off PPI session was held to discuss the acceptability and accessibility of investigating equity considerations in the PCC and the appropriateness of using the PROGRESS-Plus framework to do so.

Protocol and registration

This protocol was reported according to the Preferred Reporting Items for Systematic Review and Meta-Analysis Protocols (PRIMSA-P) 2015 statement[30] (appendix 1) and has been registered with the PROSPERO international prospective register of systematic reviews

For peer review only - http://bmjopen.bmj.com/site/about/guidelines.xhtml

(Prospero registration number: CRD42020169779, available at: https://www.crd.york.ac.uk/prospero/display_record.php?RecordID=169779).

The review will be conducted and reported in accordance with the PRISMA statement guidelines[31] and the PRISMA-E 2012 equity extension[32].

Eligibility criteria

To be included in the review, papers will have to meet the following Population, Intervention, Context, Outcomes and Study design elements:

1. Population

Inclusion criteria: People resident in high-income countries with a national PrEP provision programme (i.e., where the national governing body has approved PrEP for use and it is available from health care facilities and/or bespoke initiatives) or demonstration/implementation project, regardless of sexual orientation/identity, age, gender identity, or occupation.

Exclusion criteria: People resident in countries without a national PrEP provision program or demonstration/implementation project; people resident in low- or middle-income countries.

2. Intervention

The issue to be reviewed is outcome measures for five stages of the PCC (awareness, acceptability, uptake, adherence and retention). Definitions of outcome measures for the PCC in real-world settings will be collated and reviewed, rather than the reported outcomes. For example, in studies reporting PrEP awareness, the definition of awareness and the metrics used to determine awareness are relevant to this review, whereas the reported level of awareness is not. As such, studies which include descriptions of how the main stages of the PCC will be evaluated but do not report these outcomes (e.g. study protocols) will be included.

Inclusion criteria: Studies reporting an outcome measure definition for at least one stage of the PCC in a real-world setting.

Exclusion Criteria: Studies that do not report an outcome measure definition for the PCC, randomised controlled trials (RCTs).

3. Context

Inclusion criteria: Studies conducted in high income countries, as defined by the World Bank country classifications[33]. Inequity is a relative concept, and inequities experienced by vulnerable populations in lower and middle-income countries may differ from that experienced by vulnerable populations in high-income countries with well-resourced healthcare settings.

Exclusion criteria: Studies conducted in low- or middle- income countries.

4. Outcomes

For peer review only - http://bmjopen.bmj.com/site/about/guidelines.xhtml

Primary outcomes: Studies will be included if they report at least one outcome measure definition for the PCC relating to PrEP provision in a real-world setting, i.e. an implementation trial, demonstration project or national programme. Studies only reporting absolute numbers for stages of the PCC will not be included.

Secondary outcomes: Data on whether factors influencing health equity (defined by the PROGRESS-Plus framework) are considered for the description of baseline characteristics of participants and/or when reporting measurable outcomes for the PCC.

5. Study Design

Inclusion criteria: Quantitative research studies including non-experimental observational studies (e.g. cohort, cross-sectional, and longitudinal studies) and abstracts from key international HIV/STI conferences (e.g. HIV/STI World Congress, International AIDS society, Conference on Retroviruses and Opportunistic Infections). Mixed method studies will be included providing a quantified outcome measure of the PCC is reported. Studies resulting from demonstration and/or implementation trials will also be included. Study protocols, policy frameworks and clinical guidelines will be included if they include definitions of outcome measures in relation to a real-world programme; for example definitions for how the numerator (e.g. the number of people with high adherence to PrEP) and denominator (e.g. the number of people taking PrEP) will be defined in a measure of PrEP adherence.

Exclusion Criteria: Qualitative studies, , review articles, case studies, studies not involving oral emtricitabine/tenofovir disoproxil fumarate as PrEP, and commentary or opinion pieces that do not contain a definition for an outcome measure of the PCC. Randomised controlled trials (RCTs) will also be excluded, as RCTs in high income countries predominately focus on safety and efficacy, rather than outcome measures of the PCC.

Information sources

Electronic searches

To identify studies, the following electronic databases will be searched: MEDLINE (via Ovid), EMBASE (via Ovid), PubMed, Cumulative Index to Nursing and Allied Health Literature (CINAHL) (via EBSCO Host), and Applied Social Sciences Index and Abstracts (ASSIA) (via ProQuest).

Search strategy

Restrictions on English language articles as well as for articles published between January 1st, 2012 (the year the United States Federal Drug Administration approved Truvada [emtricitabine/tenofovir disoproxil] fumarate for PrEP use[34]) and March 3rd, 2020 will be applied. The searches will be re-run prior to the final analyses and further studies retrieved for inclusion.

Medical Subject headings (MeSH), subject headings and keyword lists will be created by using language that describes 1) oral PrEP and 2) the following outcome measures of the PCC: awareness, acceptability, uptake, adherence and retention. Boolean combinations will

For peer review only - http://bmjopen.bmj.com/site/about/guidelines.xhtml

be employed to refine the search strategy. Several key articles will be identified in the preliminary searches and used to test the sensitivity of the search strategy.

The search strategy that will be used to search MEDLINE (via the Ovid platform) is presented in appendix 2. Search terms will be modified for databases where the indexing of subject headings differs from the terms used in MEDLINE.

The references of articles meeting the inclusion criteria will be searched to identify any relevant papers missed in the electronic searches.

Study selection

All citations retrieved from the electronic searches will be imported into an EndNote database. After duplicates are removed, the remaining studies will be screened by title and abstract by one reviewer, with a random sample independently reviewed by a second reviewer. Reviewers will compare their decisions throughout the process and discuss all references where there was a disagreement. A third reviewer will be asked to adjudicate where discrepancies cannot be resolved. Studies remaining after the initial title and abstract screen will undergo full text screening. Following the full-text screening, data will be extracted from all studies that meet the inclusion criteria. A flow diagram will be produced to show selection process, according to PRISMA guidelines[35], with explanation for those excluded at each stage.

Data extraction and management

Data will be extracted into a pilot-tested, standardised form by one reviewer with a random sample checked by a second reviewer. Any discrepancies will be resolved by discussion or adjudication by a third reviewer where necessary.

All studies that meet the inclusion criteria will be described in terms of:

- Bibliographic information (first author, year of study, country of study)
- Study aims and design (including study population, sample size, and data collection methods)
- Participants, demographic characteristics and number of baseline PROGRESS-Plus equity characteristics reported
- Outcome measure definitions (for PrEP awareness, acceptability, uptake, adherence, and retention).
- Whether the study considered PROGRESS-Plus equity characteristics when measuring outcomes of the PCC.

For studies that consider PROGRESS-Plus equity characteristics in outcome measures, further data extraction detailing how the PROGRESS-Plus characteristics are expressed, and additional information on the study methods and study results will be extracted into an expanded data collection form.

Risk of bias assessment of included studies

For peer review only - http://bmjopen.bmj.com/site/about/guidelines.xhtml

The quality of each paper included in the data extraction stage will be assessed independently by two reviewers, using the Effective Public Health Practice Project (EPHPP) "Quality Assessment Tool for Quantitative Studies"[36]. Individual studies will be categorised as "strong", "moderate", or "weak" in the following domains: selection bias, study design, confounders, blinding, data collection method, and withdrawals and drop outs. Studies will also be given a global score to indicate overall quality. Any discrepancies between the two reviewers will be resolved by consensus, or by a third reviewer if necessary.

Data synthesis and analysis

A narrative synthesis of results will be conducted. Individual study characteristics and outcomes will be summarised and presented in an evidence table.

To assess the extent to which studies consider the effects of PROGRESS-Plus equity characteristics in the outcome measures of the PCC, a two-stage analysis will be carried out. At the first stage, data will be presented on PCC outcome measure definitions and whether the study considers any of the PROGRESS-Plus equity characteristics for the description of baseline characteristics of study participants. The second stage we will describe whether the study considered PROGRESS-Plus characteristics in outcome measures of the PrEP continuum, and which methods were used (e.g. consideration as confounders by adjusting in multivariable analysis, as effect modifiers in stratified analysis, using interaction terms in multivariable analysis etc.). The ways in which inequities are expressed (i.e. whether absolute or relative) will also be reported, as the decision about whether to measure inequity in relative or absolute terms may influence their interpretation.

DISCUSSION

To the best of the author's knowledge, this is the first systematic review to collate definitions for outcome measures of the PCC and describe the extent to (and ways in which) equity characteristics are considered in these outcomes. The findings will have relevance to healthcare professionals, policymakers and commissioners in informing how to evaluate PrEP implementation programmes, and how to view these evaluations through an equity lens to optimise HIV prevention strategies and inform new implementation strategies for vulnerable and less advantaged populations.

Ethics and Dissemination

Ethical approval is not required for this study. When complete, the review results will be submitted for publication in a peer-reviewed journal and submitted to be presented at national and international conferences (where eligible). The findings, dissemination strategy, and lay summary will be discussed with a PPI panel.

Authors contributions

MC and LMD designed the study. MC developed and refined the study protocol with contributions from all co-authors (DS, JS, GR, HM and LMD). MC prepared the manuscript. MC will undertake data collection (literature search, data extraction), analysis,

For peer review only - http://bmjopen.bmj.com/site/about/guidelines.xhtml

interpretation and report writing. All co-investigators will contribute to the design, analysis, interpretation and report writing. All authors read and approved the final manuscript. The authors would like to thank the PPI representative for their contributions to the research question and study design.

Funding Statement

MC is funded by the Medical Research Council Doctoral Training Partnership (grant number: MR/N013867/1). The research is funded by the National Institute for Health Research Health Protection Research Unit (NIHR HPRU) in Blood Borne and Sexually Transmitted Infections at University College London in partnership with Public Health England (reference number: NIHR200911). The views expressed in this publication are those of the authors and not necessarily those of the NHS, the Medical Research Council, the National Institute for Health Research, the Department of Health or Public Health England.

Competing interests statement:

None declared

REFERENCES

1. World Health Organization. *Guideline on When To Start Antiretroviral Therapy and on Pre-Exposure Prophylaxis for HIV*. (2015).

2. Grant, R. M. *et al.* Preexposure Chemoprophylaxis for HIV Prevention in Men Who Have Sex with Men. *N. Engl. J. Med.* **363**, 2587–2599 (2010).

3. Baeten, J. M. *et al.* Antiretroviral Prophylaxis for HIV Prevention in Heterosexual Men and Women. *N. Engl. J. Med.* **367**, 399–410 (2012).

4. Country Updates – PrEPWatch. https://www.prepwatch.org/in-practice/country-updates/.

5. Pyra, M. N. *et al.* Global implementation of PrEP for HIV prevention: setting expectations for impact. *J. Int. AIDS Soc.* **22**, (2019).

6. Marmot, M., Friel, S., Bell, R., Houweling, T. A. & Taylor, S. Closing the gap in a generation: health equity through action on the social determinants of health. *Lancet* **372**, 1661–1669 (2008).

7. Public Health England. Trends in new HIV diagnoses and people receiving HIV-related care in the United Kingdom: data to the end of December 2017. *Heal. Prot. Rep. Adv. Access Rep. [v2]* **volume 12**, (2018).

8. Centers for Disease Control and Prevention. HIV and Young Men Who Have Sex With Men. *Natl. Cent. HIV/AIDS, VIral Hepatitis, STD TB Prev.* 1–4 (2014).

9. Mayer, K. H., Grinsztejn, B. & El-Sadr, W. M. Transgender People and HIV Prevention. *JAIDS J. Acquir. Immune Defic. Syndr.* **72**, S207–S209 (2016).

10. Grossman, C. I., Purcell, D. W., Rotheram-Borus, M. J. & Veniegas, R. Opportunities for HIV

For peer review only - http://bmjopen.bmj.com/site/about/guidelines.xhtml

combination prevention to reduce racial and ethnic health disparities. *Am. Psychol.* **68**, 237–246 (2013).

11. UNAIDS. *Miles to go: closing gaps, breaking barriers, fighting injustices*. (2018).

12. Elifson, K. W., Sterk, C. E. & Theall, K. P. Safe living: The impact of unstable housing conditions on HIV risk reduction among female drug users. *AIDS Behav.* **11**, (2007).

13. Pellowski, J. A., Kalichman, S. C., Matthews, K. A. & Adler, N. A pandemic of the poor: social disadvantage and the U.S. HIV epidemic. *Am. Psychol.* **68**, 197–209.

14. Rapid Response Service. *Intersectionality in HIV and Other Health-Related Research*. www.health.gov.on.ca/en/public/ (2013).

15. Collins, P. Y., von Unger, H. & Armbrister, A. Church ladies, good girls, and locas: Stigma and the intersection of gender, ethnicity, mental illness, and sexuality in relation to HIV risk. *Soc. Sci. Med.* **67**, 389–397 (2008).

16. Kelley, C. F. *et al.* Applying a PrEP Continuum of Care for Men Who Have Sex with Men in Atlanta, Georgia. *Clin. Infect. Dis.* **61**, 1590–1597 (2015).

17. Nunn, A. S. *et al.* Defining the HIV pre-exposure prophylaxis care continuum. *AIDS* **31**, 731–734 (2017).

18. Chartier, M. *et al.* Accessibility and Uptake of Pre-Exposure Prophylaxis for HIV Prevention in the Veterans Health Administration. *Fed Pr.* **35**, S42-s48 (2018).

19. South African National Department of Health (NDoH). Pre-exposure prophylaxis implementation in South Africa. in *Presented at the 19th Annual International Conference on AIDS and STIs in Africa (ICASA)* (2017).

20. PrEP Impact Trial. A pragmatic health technology assessment of PrEP and implementation. Trial Protocol. v3.0. https://www.prepimpacttrial.org.uk/protocol.

21. Dunbar, M. S. *et al.* Understanding and measuring uptake and coverage of oral pre-exposure prophylaxis delivery among adolescent girls and young women in sub-Saharan Africa. *Sexual Health* vol. 15 513–521 (2018).

22. Duff, P. *et al.* Barriers to Viral Suppression among Female Sex Workers: Role of Structural and Intimate Partner Dynamics. *J. Acquir. Immune Defic. Syndr.* **73**, 83–90 (2016).

23. Gari, S. *et al.* Access to HIV/AIDS care: A systematic review of socio-cultural determinants in low and high income countries. *BMC Health Services Research* vol. 13 (2013).

24. Yi, S. *et al.* Awareness and willingness to use HIV pre-exposure prophylaxis among men who have sex with men in low- and middle-income countries: A systematic review and meta-analysis: A. *Journal of the International AIDS Society* vol. 20 (2017).

25. Janet, J. M. *et al.* Adherence to PrEP among young men who have sex with men participating in a sexual health services demonstration project in Alameda County, California. *J Acquir Immune Defic Syndr* (2019) doi:10.1097/qai.0000000000002051.

26. Zucker, J. *et al.* Predictors of Disengagement in Care for Individuals Receiving Pre-Exposure Prophylaxis (PrEP). *J Acquir Immune Defic Syndr* (2019) doi:10.1097/qai.0000000000002054.

27. O'Neill, J. *et al.* Applying an equity lens to interventions: Using PROGRESS ensures consideration of socially stratifying factors to illuminate inequities in health. *J. Clin. Epidemiol.* **67**, 56–64 (2014).

For peer review only

For peer review only - http://bmjopen.bmj.com/site/about/guidelines.xhtml

28. Kavanagh, J., Oliver, S. & Lorenc, T. Reflections on developing and using PROGRESS-Plus. *Equity Updat.* **2**, 1–3 (2008).

29. Evans, T. & Brown, H. Road traffic crashes: operationalizing equity in the context of health sector reform. *Inj. Control Saf. Promot.* **10**, 11–2.

30. Moher, D. *et al.* Preferred reporting items for systematic review and meta-analysis protocols (PRISMA-P) 2015 statement. *Rev. Esp. Nutr. Humana y Diet.* **20**, 148–160 (2016).

31. Liberati, A. *et al.* The PRISMA statement for reporting systematic reviews and meta-analyses of studies that evaluate healthcare interventions: explanation and elaboration. *BMJ* **339**, (2009).

32. Welch, V. *et al.* PRISMA-Equity 2012 Extension: Reporting Guidelines for Systematic Reviews with a Focus on Health Equity. *PLoS Med.* **9**, (2012).

33. WorldBank. World Bank Country and Lending Groups – World Bank Data Help Desk. https://datahelpdesk.worldbank.org/knowledgebase/articles/906519 (2019).

34. FDA. Truvada for PrEP Fact Sheet : Ensuring Safe and Proper Use. 1–2 (2012).

35. Shamseer, L. *et al.* Preferred reporting items for systematic review and meta-analysis protocols (prisma-p) 2015: Elaboration and explanation. *BMJ (Online)* vol. 350 (2015).

36. Quality Assessment Tool for Quantitative Studies. https://www.ephpp.ca/quality-assessment-tool-for-quantitative-studies/.

For peer review only

For peer review only - http://bmjopen.bmj.com/site/about/guidelines.xhtml

PRISMA-P (Preferred Reporting Items for Systematic review and Meta-Analysis Protocols) 2015 checklist: recommended items to address in a systematic review protocol*

Section and topic	Item No	Checklist item
**ADMINISTRATIVE INFORMATION**		
Title:		
Identification	1a	Identify the report as a protocol of a systematic review
Update	1b	If the protocol is for an update of a previous systematic review, identify as such
Registration	2	If registered, provide the name of the registry (such as PROSPERO) and registration number
Authors:		
Contact	3a	Provide name, institutional affiliation, e-mail address of all protocol authors; provide physical mailing address of corresponding author
Contributions	3b	Describe contributions of protocol authors and identify the guarantor of the review
Amendments	4	If the protocol represents an amendment of a previously completed or published protocol, identify as such and list changes; otherwise, state plan for documenting important protocol amendments
Support:		
Sources	5a	Indicate sources of financial or other support for the review
Sponsor	5b	Provide name for the review funder and/or sponsor
Role of sponsor or funder	5c	Describe roles of funder(s), sponsor(s), and/or institution(s), if any, in developing the protocol
**INTRODUCTION**		
Rationale	6	Describe the rationale for the review in the context of what is already known
Objectives	7	Provide an explicit statement of the question(s) the review will address with reference to participants, interventions, comparators, and outcomes (PICO)
**METHODS**		
Eligibility criteria	8	Specify the study characteristics (such as PICO, study design, setting, time frame) and report characteristics (such as years considered, language, publication status) to be used as criteria for eligibility for the review
Information sources	9	Describe all intended information sources (such as electronic databases, contact with study authors, trial registers or other grey literature sources) with planned dates of coverage
Search strategy	10	Present draft of search strategy to be used for at least one electronic database, including planned limits, such that it could be repeated
Study records:		
Data management	11a	Describe the mechanism(s) that will be used to manage records and data throughout the review

For peer review only

Selection process	11b	State the process that will be used for selecting studies (such as two independent reviewers) through each phase of the review (that is, screening, eligibility and inclusion in meta-analysis)
Data collection process	11c	Describe planned method of extracting data from reports (such as piloting forms, done independently, in duplicate), any processes for obtaining and confirming data from investigators
Data items	12	List and define all variables for which data will be sought (such as PICO items, funding sources), any pre-planned data assumptions and simplifications
Outcomes and prioritization	13	List and define all outcomes for which data will be sought, including prioritization of main and additional outcomes, with rationale
Risk of bias in individual studies	14	Describe anticipated methods for assessing risk of bias of individual studies, including whether this will be done at the outcome or study level, or both; state how this information will be used in data synthesis
Data synthesis	15a	Describe criteria under which study data will be quantitatively synthesised
	15b	If data are appropriate for quantitative synthesis, describe planned summary measures, methods of handling data and methods of combining data from studies, including any planned exploration of consistency (such as I^2, Kendall's τ)
	15c	Describe any proposed additional analyses (such as sensitivity or subgroup analyses, meta-regression)
	15d	If quantitative synthesis is not appropriate, describe the type of summary planned
Meta-bias(es)	16	Specify any planned assessment of meta-bias(es) (such as publication bias across studies, selective reporting within studies)
Confidence in cumulative evidence	17	Describe how the strength of the body of evidence will be assessed (such as GRADE)

*** It is strongly recommended that this checklist be read in conjunction with the PRISMA-P Explanation and Elaboration (cite when available) for important clarification on the items. Amendments to a review protocol should be tracked and dated. The copyright for PRISMA-P (including checklist) is held by the PRISMA-P Group and is distributed under a Creative Commons Attribution Licence 4.0.**

From: Shamseer L, Moher D, Clarke M, Ghersi D, Liberati A, Petticrew M, Shekelle P, Stewart L, PRISMA-P Group. Preferred reporting items for systematic review and meta-analysis protocols (PRISMA-P) 2015: elaboration and explanation. BMJ. 2015 Jan 2;349(jan02 1):g7647.

For peer review only

Appendix 2

Systematic Review Search Strategy

1. exp HIV Infections/
2. Pre-Exposure Prophylaxis/
3. Chemoprevention/
4. Anti-HIV Agents/
5. Anti-Retroviral Agents/
6. 1 or 4 or 5
7. 2 or 3
8. 6 and 7
9. ((pre-exposure or preexposure or pre exposure) adj2 prophyla*).tw
10. PrEP.tw
11. ((pre-exposure or preexposure) adj2 chemoprophyla*).tw
12. 10 or 11
13. chemoprophylaxis.tw
14. chemo-prophylaxis.tw
15. chemoprevention.tw
16. chemo-prevention.tw
17. 12 or 13 or 14 or 15 or 16
18. truvada.tw
19. Emtricitabine/
20. Tenofovir/
21. 19 and 20
22. 18 or 21
23. (HIV or Human Immunodeficiency virus).tw
24. 17 and 23
25. 8 or 9 or 22 or 24
26. limit 25 to yr="2012 -Current"
27. limit 26 to (english language and humans)
28. exp Attitude to Health/
29. exp "Treatment Adherence and Compliance"/

For peer review only

30. Program Evaluation/mt, st, sn [Methods, Standards, Statistics & Numerical Data]

31. ((care or care-) adj2 (cascade or continuum)).tw

32. ((prevention or prevention-) adj2 (cascade or continuum)).tw

33. (non-adherence or nonadherence or non adherence).tw

34. (noncompliance or non-compliance or non compliance).tw

35. access.tw

36. adhere*.tw

37. attitude*.tw

38. aware*.tw

39. compliance.tw

40. compliant.tw

41. demand.tw

42. disengage*.tw

43. enable*.tw

44. engage*.tw

45. evaluat*.tw

46. experience*.tw

47. implement*.tw

48. interest.tw

49. knowledge.tw

50. measure*.tw

51. perception*.tw

52. retain*.tw

53. retention.tw

54. uptake.tw

55. 28 or 29 or 30 or 31 or 32 or 33 or 34 or 35 or 36 or 37 or 38 or 39 or 40 or 41 or 42 or 43 or 44 or 45 or 46 or 47 or 48 or 49 or 50 or 51 or 52 or 53 or 54

56. 27 and 55

For peer review only

Reporting checklist for protocol of a systematic review.

Based on the PRISMA-P guidelines.

Instructions to authors

Complete this checklist by entering the page numbers from your manuscript where readers will find each of the items listed below.

Your article may not currently address all the items on the checklist. Please modify your text to include the missing information. If you are certain that an item does not apply, please write "n/a" and provide a short explanation.

Upload your completed checklist as an extra file when you submit to a journal.

In your methods section, say that you used the PRISMA-Preporting guidelines, and cite them as:

Moher D, Shamseer L, Clarke M, Ghersi D, Liberati A, Petticrew M, Shekelle P, Stewart LA. Preferred Reporting Items for Systematic Review and Meta-Analysis Protocols (PRISMA-P) 2015 statement. Syst Rev. 2015;4(1):1.

		Reporting Item	Page Number
**Title**			
Identification	#1a	Identify the report as a protocol of a systematic review	pg 1, line 2
Update	#1b	If the protocol is for an update of a previous systematic review, identify as such	N/A
**Registration**			
	#2	If registered, provide the name of the registry (such as PROSPERO) and registration number	pg 6, line 200 - 201
**Authors**			
Contact	#3a	Provide name, institutional affiliation, e-mail address of all protocol authors; provide physical mailing address of corresponding author	pg1, line 4-26
Contribution	#3b	Describe contributions of protocol authors and identify the	pg10, line 347-

For peer review only

		guarantor of the review	353
**Amendments**			
	#4	If the protocol represents an amendment of a previously completed or published protocol, identify as such and list changes; otherwise, state plan for documenting important protocol amendments	N/A
**Support**			
Sources	#5a	Indicate sources of financial or other support for the review	pg 10, line 355 - 361
Sponsor	#5b	Provide name for the review funder and / or sponsor	N/A
Role of sponsor or funder	#5c	Describe roles of funder(s), sponsor(s), and / or institution(s), if any, in developing the protocol	N/A
**Introduction**			
Rationale	#6	Describe the rationale for the review in the context of what is already known	pg 4-5, line 103 -170
Objectives	#7	Provide an explicit statement of the question(s) the review will address with reference to participants, interventions, comparators, and outcomes (PICO)	pg. 5-6, line 178 - 189
**Methods**			
Eligibility criteria	#8	Specify the study characteristics (such as PICO, study design, setting, time frame) and report characteristics (such as years considered, language, publication status) to be used as criteria for eligibility for the review	pg. 6-7, line 207-250
Information sources	#9	Describe all intended information sources (such as electronic databases, contact with study authors, trial registers or other grey literature sources) with planned dates of coverage	pg. 8, line 261 - 280
Search strategy	#10	Present draft of search strategy to be used for at least one electronic database, including planned limits, such that it could be repeated	pg. 8, line 276 – 278(and appendix 2)
Study records - data management	#11a	Describe the mechanism(s) that will be used to manage records and data throughout the review	pg. 8, line 282 – 283

For peer review only

Study records - selection process	#11b	State the process that will be used for selecting studies (such as two independent reviewers) through each phase of the review (that is, screening, eligibility and inclusion in meta-analysis)	pg. 8-9, line 282 – 291
Study records - data collection process	#11c	Describe planned method of extracting data from reports (such as piloting forms, done independently, in duplicate), any processes for obtaining and confirming data from investigators	pg. 9, line 292 – 295
Data items	#12	List and define all variables for which data will be sought (such as PICO items, funding sources), any pre-planned data assumptions and simplifications	pg. 9, line 296 – 309
Outcomes and prioritization	#13	List and define all outcomes for which data will be sought, including prioritization of main and additional outcomes, with rationale	pg. 7, line 237 – 244
Risk of bias in individual studies	#14	Describe anticipated methods for assessing risk of bias of individual studies, including whether this will be done at the outcome or study level, or both; state how this information will be used in data synthesis	pg. 9, line 310 – 317
Data synthesis	#15a	Describe criteria under which study data will be quantitatively synthesised	N/A
Data synthesis	#15b	If data are appropriate for quantitative synthesis, describe planned summary measures, methods of handling data and methods of combining data from studies, including any planned exploration of consistency (such as I2, Kendall's τ)	N/A
Data synthesis	#15c	Describe any proposed additional analyses (such as sensitivity or subgroup analyses, meta-regression)	N/A
Data synthesis	#15d	If quantitative synthesis is not appropriate, describe the type of summary planned	pg. 9, line 318 – 331
Meta-bias(es)	#16	Specify any planned assessment of meta-bias(es) (such as publication bias across studies, selective reporting within studies)	N/A
Confidence in cumulative	#17	Describe how the strength of the body of evidence will be assessed (such as GRADE)	pg. 9, 310 – 317

For peer review only

evidence

The PRISMA-P checklist is distributed under the terms of the Creative Commons Attribution License CC-BY 4.0. This checklist was completed on 19. May 2020 using https://www.goodreports.org/, a tool made by the EQUATOR Network in collaboration with Penelope.ai

For peer review only
